# A new antimicrobial PVC-based polymeric material incorporating bisacylthiourea complexes

**DOI:** 10.1186/s13065-023-00958-7

**Published:** 2023-05-03

**Authors:** Hammed H. A. M. Hassan, Amel F. ELhusseiny

**Affiliations:** Chemistry Department, Faculty of Science, Alexandria University, P.O. Box 2-Moharam Bek, Alexandria, 21568 Egypt

**Keywords:** Antimicrobial, Complexes, Composite, PVC polymer, Thiourea, Surface morphology, Thermal

## Abstract

A new antimicrobial material incorporating Cu(I) and Cd(II) complexes of bisacylthiourea derivatives in a PVC film was successfully synthesized and characterized by IR, UV, NMR, SEM, and thermal analyses. The results revealed that on coordination, the electronic structure change of the ligand affects practically all their spectral vibrational pattern; however, within the complex pattern, some vibrations indicated that the thiourea derivative behaves as a neutral ligand, which coordinates the metal ion through the sulfur atom of the thiocarbonyl group. The greater affinity of the S atom for Cu^+ 1^ played a role in Cu(II)→Cu(I) reduction, and the intramolecular hydrogen bonds of the type of (NH···Cl) further stabilized the obtained Cu(I) complex in dioxane. The antimicrobial activity shows that all investigated compounds exhibit excellent activity compared to standard antibiotics. The antibacterial power of the PVC/Cd composite is significantly superior against the most resistant species to both disinfectants and antibiotics compared to its PVC/Cu analogue; nevertheless, the latter exhibited activity equal to an average halo diameter of 29 ± 0.33 mm against pathogenic *E. coli ATCC 25,922*, indicating excellent G (-) activity. Interestingly, the PVC/Cd composite exhibited excellent activity against pathogenic *C. albicans RCMB 005003 (1) ATCC 10,231*, while its PVC/Cu analogue was inactive. These materials may be used to reduce infection in wounds either as a composite film or coated barrier dressings, and in addition, the results should open a new direction in antimicrobial surface engineering within the biomedical field. Further challenges are the development of reusable and broad-range antimicrobial polymers.

.

**Supplementary Information:**

The online version contains supplementary material available at 10.1186/s13065-023-00958-7.

## Introduction

The current pandemic crisis has found an urgent need to face the problem of the introduction and spread of evolving pathogens into the environment that produce various types of contamination. One of them is surface microbial contamination, a main task in the fields of consumer protection, human healthcare, and food safety around the world [1]. The gravity of infections is maximized during microbial surface attachment, and growth occurs in a self-formed biofilm [2], which is considered a source of infection and signifies a pool of microorganisms. In this regard, the surface engineering of materials with antibacterial properties and their mode of action is considered one of the possible ways to increase biocompatibility and thereby reduce the danger of microbial contamination and avoid biofilm infection by modifying surfaces and material properties [3–12]. The functionalization of polymers with antimicrobial agents has gained considerable interest for the development of biomaterials capable of retarding microbial colonization and preventing the spread of pathogenic microorganisms [13, 14]. Biocide-releasing polymers are an efficient class of antimicrobial polymers that involve polymers loaded with biocide molecules [15].

Poly(vinyl chloride) (PVC) is a widely used commercial thermoplastic polymer and one of the most widely used surfaces in the medical field. Modification of PVC is currently being studied as an antimicrobial material, as it would reduce the risk of infections and cross-contamination in industrial processes [16, 17]. However, modified polymers with an active organic and/or inorganic admixture can produce a target compound that can alter the cell walls of microorganisms and destroy the cytoplasmic membrane by forming a complex with the lipid bilayer [18]. In this sense, nisin and triclosan antibiotics were successfully incorporated into PVC products [19, 20]. Silver/PVC nanocomposites proved to be an efficient antibacterial agent [21] using either ionic or metallic silver [22]. Evaluation of PVC compounds containing different doses of silver, zinc and zeolite clearly pointed out the formation of bacterial biofilms as the main factor of the antimicrobial effect of the additive [23]. Furthermore, an efficient antimicrobial powder based on guanidine, symmetrical porphyrin PVC plastics [24, 25], and maleimido phenyl thiourea composite-based PVC powder [26] were reported.

Substituted thiocarbamides are rich sources of nitrogen and sulfur atoms and have long been known as important compounds in academia and industry. These compounds have gained importance for their use in the synthesis of several biologically active heterocyclic compounds, and their coordination chemistry has potential applications in various areas. The presence of nitrogen and sulfur donor atoms provides a multitude of bonding possibilities, enabling them to act as versatile ligands that can coordinate the metal centers as neutral, mono, or di-anionic groups to form stable complexes that have applications such as biological, corrosion inhibitors, catalysts, sensors, optical, precursors in polymerization processes, plant protection agents, and pesticides. The existence of two units of reactive primary amine groups has made these derivatives suitable precursors for the synthesis of many of their derivatives, which have displayed a broad range of applications in the pharmaceutical industry because of their antiparasitic, anticancer, antioxidant, antibacterial, antifungal, and anti-HIV properties. In addition, these compounds have been reported to possess herbicidal, insecticidal, antituberculosis, and anticonvulsant properties. The coordination compounds of thioureas have received tremendous attention due to their anti-inflammatory, antimalarial, antimicrobial, and antitumour activities [27].

Phenylthiourea derivatives have been proven to have great biological properties, including insecticide herbicidal or bactericidal properties [28–32], and the nature of the substituent is critical for their activities [33]. Arylthioureas have (O, N, S) potential donors and have been considered model compounds for various intermolecular and intramolecular interactions involving sulfur [34]. Hypodentate is a monodentate ligand that coordinates only through the sulfur atom with many metal ions to form the corresponding metal complex [35, 36]. The use of metal complexes with excellent cytotoxic activity has been reported [37], where the complex significantly improved the biological results obtained with the pure thiourea ligand. Variations of the thioureas and/or their complexes have the potential to become novel metal-based drugs, as evidenced by the recently discovered potent anticancer thiourea derivatives [38–41]. The NH moieties of these ligands favor the formation of hydrogen bonds, which can increase their aqueous-medium solubility and allow cellular acceptance. Furthermore, the orientation of the sulfur-donor atom in the backbone of thiourea permits it to bond to transition metal ions in a square-planar geometry. It has been reported that numerous stable metal complexes of thioureas have interesting physical and chemical properties and important biological activity [35, 36].

In view of the above, the substituted and unsubstituted biphenyl thiourea substituents exhibited interesting biological data in most reported strategies directed to the discovery of new lead antibacterial compounds [34]. For this reason, it is interesting to develop a new antimicrobial plastic material that incorporates transition metal complexes of the oxybis (4,1) phenylene moiety flanked symmetrically by two phenylthioureas in a PVC film. It should be noted that new antibacterial plastic materials can be developed using chemical modification and/or polymer mixing [42, 43]. The most widely used method of preparing antibacterial plastics is the incorporation of many inorganic or organic additives into the existing polymer structure to obtain strong antibacterial and antimicrobial activities [44, 45]. In the current study, we report the synthesis, characterization, and evaluation of the antibacterial and antifungal activities of Cu(I) and Cd(II) complexes derived from N,N’-(((oxybis(4,1-phenylene))bis(azanediyl))bis (carbonothioyl))dibenzamide ligands. Cadmium and copper complexes show noticeable similarity in their structures, as they form clusters, dinuclear, or polynuclear complexes, while other complexes are monomeric [46]. The next step is to incorporate these complexes into PVC films to obtain thermally stable antimicrobial plastic that may be used to reduce infection in wounds either as a composite film or phenyl thiourea-coated barrier dressings. Several studies have verified that the incorporation of antimicrobial agents into edible films and coatings could be effective for diminishing the levels of pathogenic organisms, including gram-negative and gram-positive bacteria [47].

## Materials and methods

### Materials

Benzoyl chloride, ammonium thiocyanate (May&Baker, Dagenham, England), oxydianiline (Aldrich), 1,4-dioxane (Aldrich), dimethylsulfoxide (DMSO) and ethanol (Aldrich). Acetonitrile (BDH-PROLABO, UK) and diethyl ether (Sigma‒Aldrich, Germany) were used without further purification. CuCl_2_·2H_2_O and CdCl2·2H2O were obtained from ADWIC (Alexandria, Egypt) and used as received. PVC was obtained from Elmasrya Plastic Technology (ECPT) Co., Ltd., (Egypt).

### Measurements

Infrared spectra (IR, KBr pellets; 3 mm thickness) were recorded on a Perkin-Elmer Infrared Spectrophotometer (FTIR 1650). All spectra were recorded within the wavenumber range of 4000–600 cm^− 1^ at 25 °C. Absorption spectra were measured with a UV 500 UV‒Vis spectrometer at room temperature (rt) in DMSO with a polymer concentration of 2 mg/10 mL. Elemental analyses were performed at the Microanalytical Unit, Cairo University. The NMR spectra were recorded at the NMR unit of Mansoura University on a JEOL ECA-500 II spectrometer in DMSO-d6 solution with TMS as an internal standard. Thermogravimetric (TG) and differential thermogravimetric (DTG) analyses were carried out in the temperature range from 20 to 400 °C in a steam of nitrogen atmosphere by a Shimadzu DTG 60 H thermal analyser. The experimental conditions were platinum crucible, nitrogen atmosphere with a 30 mL/min flow rate and a heating rate of 10 C/min. Differential scanning calorimetry (DSC-TGA) analyses were carried out using SDT-Q600-V20.5-Build-15 at the microanalytical unit, Cairo University. The polymer morphologies were observed by scanning electron microscopy (SEM) (JEOL-JSMIT 200) and transmission electron microscopy (TEM) (JEOL-JTM-1400 plus) at the E-Microscope Unit, Faculty of Science, Alexandria University. The samples were sonicated in deionized water for 5 min, deposited onto carbon-coated copper mesh and allowed to air-dry before examination. Antimicrobial screening tests were performed at the Regional Center of Mycology and Biotechnology, El-Azhar University, Cairo, Egypt.

### Synthesis of N,N’-((oxybis (4,1-phenylene))bis(azanediyl))bis(carbonothioyl)) dibenzamide 4

A stirred mixture of PhCOCl **1** (6 g, 0.042 mol) and ammonium thiocyanate **2** (3.2 g, 0.042 mol) in CH_3_CN (40 ml) was refluxed for 1 h, and then a solution of oxydianiline **3** (4.20 g, 0.021 mol) dissolved in CH_3_CN (10 ml) was slowly added for 10 minutes. Reflux was continued for another 4 h, the solution was poured into cold water (200 ml), and the pale yellow ppt was filtered and recrystallized by THF/EtOH (3:1 v/v); colorless crystals, yield 82%, mp 225 °C. Calculated for C_28_H_22_N_4_O_3_S_2_; C, 63.86; H, 4.21; N, 10.64; S, 12.18; Found: C, 64.79; H, 4.08; N, 10.50; S, 11.78. IR: u 3786, 3724 (O-H), 3422 (O-H), 3241(N-H), 3118 (N’-H), 3030 (C-H_arom_), 2473, 2085, 1672 (C = O), 1599, 1528 (N’-H, thioamide band I), 1331, 1259 (C = S), 1224, 1155, 1078, 1018, 940, 841, 756, 699, 623, 507, 451. UV (l_max_, nm): 225, E = 5.51 eV, 276, E = 4.49 eV, 322, E = 3.85 eV. ^1^H-nmr (d ppm, J Hz): 12.54 (s, 2H, 2x NH), 11.59 (s, 2H, 2x NH), 7.97 (d, 4H, J 8 Hz, H-C3, H-C5, H-C3’, H-C5’), 7.66 (m, 6 H, H-C11, H-C12, H-C13, H-C11’, H-C12’, H-C13’), 7.53 (2d, 4 H, J 8 Hz, 7.5 Hz, H-C10, H-C14, H-C10’, H-C14’), 7.09 (d, 4 H, J 8.5 Hz, H-C2, H-C6, H-C2’, H-C6’). ^13^ C-nmr (d, ppm): 179.24 (2x CS), 168.25 (2x CO), 154.52 (C-1, C-1’), 133.58 (C-9, C-9’), 133.14 (C-4, C-4’), 132.15 (C-11, C-11’), 128.69 (C-13, C-13’), 128.58 (C-10, C-10’), 128.45 (C-14, C-14’), 128.29 (C-3, C-3’), 126.35 (C-5, C-5’), 121.76 (C-2), 119.75 (C-2’), 118.68 (C-7), 117.89 (C-7’).

### Metal complex Synthesis 5, 6 (General method)

A solution (15 ml) of metal chloride (CuCl_2_.2H_2_O or CdCl_2_.2H_2_O) (5 mmol) in dioxane was added to dissolved ligand **3** (5.26 g, 10 mmol) in dioxane (20 ml) and refluxed for 1 h. The obtained precipitate was filtered, thoroughly washed using EtOH and Et_2_O, and dried in a vacuum oven.

#### Bis[N,N’-(((oxybis(4,1-phenylene))bis(azanediyl)) -µ-bis(carbonothioyl))dibenzamide]di[copper(I) chloride]dihydrate 5

Yellow solid, yield 74%, m.p. 240 °C. Calculated for C_56_H_52_N_8_O_8_S_4_Cu_2_Cl_2_.2H_2_O; C, 52.09; H, 4.06; N, 8.68; S, 9.91; Found: C, 52.21; H, 3.64; N, 8.27; S, 9.17. UV (l_max_, nm): 276, E = 4.49 eV, 321, E = 3.86 eV. IR: 3919, 3884, 3849, 3783, 3700, 3424 (OH), 3234 (NH), 3139 (N’-H), 3032 (CH), 2927, 2854, 2288, 1823, 1718, 1672 (CH = O), 1601, 1528, 1502, 1324, 1257 (CH = S), 1155, 1076, 1021, 940, 842, 698, 603, 505, 453, 440. ^1^H-nmr (d ppm, J Hz): 10.95 (s, 2H, 2x NH), 10.85 (s, 2H, 2x NH), 8.91–7.80 (m, 4H, H-C3, H-C5, H-C3’, H-C5’), 7.66–7.53 (m, 10 H, H-C11, H-C12, H-C13, H-C11’, H-C12’, H-C13’, H-C10, H-C14, H-C10’, H-C14’), 7.20–6.85 (m, 4 H, H-C2, H-C6, H-C2’, H-C6’). ^13^ C-nmr (d, ppm): 179.24 (2x CS), 168.25 (2x CO), 154.52 (C-1, C-1’), 133.58 (C-9, C-9’), 133.14 (C-4, C-4’), 132.78 (C-11, C-11’), 129.19 (C-13, C-13’), 128.50 (C-10, C-10’), 128.22 (C-14, C-14’), 120.79 (C-3, C-3’), 120.76 (C-5, C-5’), 120.73 (C-2), 120.68.

#### Bis[N,N’-((oxybis (4,1-phenylene))bis(azanediyl))-m-bis(carbonothioyl))dibenzamide]di[cadmium (II) chloride] 6

Solid white, yield 76%, mp ≥ 300 °C. Calculated for C_56_H_44_N_8_O_6_S_4_Cd_2_Cl_4_; C, 47.24; H, 3.40; N, 7.87; S, 9.01; Found: C, 47.69; H, 3.38; N, 7.42; S, 8.09. UV (l_max_, nm): 275, E = 4.51 eV, 320, E = 3.87 eV. IR: 3630.49, 3538.38, 3213.74, 3143.11, 3027.37, 1900.23, 1661.82, 1595.23, 1528.26, 1329.70, 1270.53, 1227.12, 1157.24, 1111.71, 1021.00, 943.17, 848.33, 802.87, 752.96, 716.01, 675.41, 601.43, 507.37, 454.91. ^1^H-nmr (d ppm, J Hz): 12.56 (s, 2H, 2x NH), 11.52 (s, 2H, 2x NH), 7.99 (d, 4H, J 7.2 Hz, H-C3, H-C5, H-C3’, H-C5’), 7.93 (m, 6 H, H-C11, H-C12, H-C13, H-C11’, H-C12’, H-C13’), 7.68 (2d, 4 H, J 8 Hz, 7.5 Hz, H-C10, H-C14, H-C10’, H-C14’), 7.36 (d, 4 H, J 8.5 Hz, H-C2, H-C6, H-C2’, H-C6’). ^13^ C-nmr (d, ppm): 179.19 (2x CS), 168.21 (2x CO), 154.48 (C-1, C-1’), 133.55 (C-9, C-9’), 133.09 (C-4, C-4’), 132.78, 132.09 (C-11, C-11’), 129.20, 128.61 (C-13, C-13’), 128.49 (C-10, C-10’), 128.40 (C-14, C-14’), 126.24 (C-5, C-5’), 119.55 (C-2’), 118.87 (C-7), 118.63 (C-7’).

### Preparation of biologically active PVC composite films (general method) [48]

The PVC/nanocomposite films were synthesized by solution casting as follows: 0.5 g of PVC dissolved in THF (10 ml) was stirred magnetically at rt for 30 min to achieve homogeneity, then ligand **4** or metal nanocomposites **5** and **6** (∼20 mg) dissolved in THF (5 ml) was added to the PVC solution and stirred for another 30 min. Finally, the obtained mixture was cast onto glass Petri dishes and dried at room temperature. In a similar fashion, the neat PVC film was prepared as a control without adding any additional additives. The average film thickness was 200 mm, and the incorporation of additives **4**–**6** did not alter the thickness. The following spectroscopic data were recorded: PVC: UV (l_max_, nm): 255, band gap (E) = 4.86 eV. IR (u, cm^− 1^): 3818, 3334, 2949, 2888, 2733, 2364, 2208, 2063, 1964, 1767, 1721, 1649, 1437, 1348, 1189, 1063, 1034, 1005, 922, 855, 750, 692, 635, 562, 474, 431. PVC/**4** composite: UV (l_max_, nm): 260, E = 4.77 eV, 265 (sh), E = 4.68 eV, 302, E = 4.11 eV. IR (u, cm^− 1^): 3655, 3427, 2910, 2727, 2365, 2278, 1947, 1771, 1723, 1685, 1608, 1547, 1434, 1329, 1252, 1199, 1104, 1032, 921, 839, 695, 611, 491, 426. PVC/**5** composite: UV (l_max_, nm): 260, E = 4.77 eV, 265 (sh), E = 4.68 eV, 300, E = 4.13 eV. IR (u, cm^− 1^): 3520, 3362, 2921, 2863, 2727, 2369, 2045, 1953, 1772, 1732, 1624, 1453, 1367, 1276, 1109, 1033, 912, 702, 645, 492, 428. PVC/**6** composite: UV (l_max_, nm): 260, E = 4.77 eV, 265 (sh), E = 4.68 eV, 300, E = 4.13 eV. IR (u, cm^− 1^): 3840, 3424, 2914, 2727, 1973, 1771, 1722, 1679, 1604, 1536, 1499, 1432, 1331, 1261, 1194, 1102, 1033, 922, 840, 694, 610, 493, 426.

### Antimicrobial activity

The antimicrobial activity of organic ligand **4** and its complexes **5** and **6** was tested against gram-positive bacterial strains (*S. pneumoniae RCMB 010010* and *B. subtilis RCMB 010067*), gram-negative bacteria (*E. coli RCMB 010052* and *P. aeruginosa RCMB 010043*) and pathogenic fungi (*C. albicans RCMB 05036* and *(A) fumigatus RCMB 02568*) using DMSO as a negative control. Gentamicin, ampicillin, and amphotericin B were used as antimicrobial and antifungal positive controls. The antimicrobial activity of the examined samples was investigated by the modified Kirby-Bauer disc diffusion method [49]. All the prepared compounds were dissolved to synthesize a stock solution of 1 mg/mL using DMSO. The stock solution was aseptically transferred and diluted twofold to obtain solutions of various concentrations. Antibacterial and antifungal activities were determined using the filter paper disc method [50], and activities were determined by measuring the diameters of the inhibition zone (mm). The medium with DMF was used as a control. All cultures were kept on nutrient agar (NA) and incubated at 37 °C. The inoculums of bacteria were achieved by growing the culture in NA broth at 37 °C overnight. Approximately 0.1 mL of diluted bacterial or fungal culture suspension was spread on NA plates uniformly. Solutions of the tested compounds and reference drugs were prepared by dissolving 10 mg of the compound in 10 ml of DMF. A 100 µL volume of each sample was pipetted into a hole (depth 3 mm) made in the center of the agar. Sterile 8 mm discs (Himedia Pvt. Ltd.) were permeated with test compounds. The disc was located on the plate. Each plate had one control disc soaked with solvent. The plates were incubated at 37 °C for 18–48 h. Standard discs of tetracycline (antibacterial agent; 10 µg/disc) and amphotericin B (antifungal agent; 10 µg/disc) served as positive controls for antimicrobial activity, while filter discs soaked with 10 µL of solvent DMSO were utilized as a negative control. All experiments were performed at least in triplicate, and the outcomes were averaged. The antimicrobial activities of the synthesized composite films PVC, PVC/**4**, PVC/**5** and PVC/**6** were evaluated against *S. aureus ATCC 25,923, (B) subtilis RCMB 015 (1) NRRL B-543, E. coli ATCC 25,922, P. aeruginosa ATCC 27,853, (C) albicans RCMB 005003 (1) ATCC 10,231*, and *A. fumigatus RCMB 002008*. Gentamicin and ketoconazole B were utilized as antimicrobial and antifungal controls, respectively. Susceptibility tests were accomplished according to the recommendations of NCCLS (National Committee for Clinical Laboratory Standards, 1993). Screening tests of the inhibition zone were carried out using the disk diffusion method [51]. The inoculum suspension was prepared from colonies grown overnight on an agar plate and inoculated into Mueller-Hinton broth (fungi using malt broth). A sterile pad was immersed in the suspension and used to inoculate Mueller-Hinton agar plates (fungi using malt agar plates and bacteria using nutrient agar plates). The disks of compounds are added to the surface of the agar. The inhibition zone was measured around each disk after 48 h at 28 °C for fungi and 24 h at 37 °C for bacteria.

## Results and discussion

**Scheme 1:**
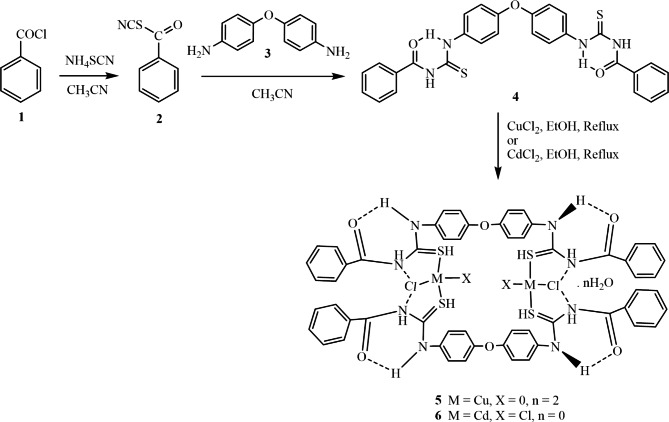
Chemical synthesis of bis-phenylthiourea derivative **4** and its metal complexes **5** and **6**

As shown in Scheme 1, a mixture of equimolar ratios of benzoyl chloride **1** and ammonium thiocyanate **2** in acetonitrile was heated under reflux for 1 h, followed by dropwise addition of a one-half equivalent ratio of oxydianiline **3** dissolved in acetonitrile. Ligand **4** was obtained as colorless crystals with a high yield of 82%. Reaction of equimolar amounts of ligand **4** with copper (II) or cadmium (II) chlorides provided the complex [Cu_2_(H_2_L)_2_Cl_2_]·nH_2_O **5** and [Cd_2_(H_2_L)_2_Cl_4_]·nH_2_O **6**, where n = 2 or 0, respectively. Structures of ligand **4** and its copper and cadmium complexes **5** and **6** were fully characterized by various analytical methods as subsequently discussed henceforth. It should be noted that the greater affinity of S for Cu^+ 1^ appears to play a role in Cu(II)→Cu(I) reduction. This assumption could be explained by the oxidation of the benzoyl thiourea derivative to disulfide, while Cu^+ 2^ is reduced to Cu^+ 1^ and remains in the medium as a complex with acetonitrile [52]. At the same time, intramolecular hydrogen bonds of the type of (NH···Cl) may stabilize the obtained Cu(I) complex [53]. The PVC/nanocomposite films PVC/**4** to PVC/**6** were prepared by solution casting by dissolving 0.5 g of PVC in THF (10 ml) followed by the addition of **4**–**6** (∼10 mg) dissolved in THF (5 ml). The neat PVC film was prepared as a control without adding any additional additives in a similar procedure. The structures and properties of the prepared film composites were fully characterized by various methods, as discussed below.

### FT-IR spectroscopy

The FT-IR spectrum of ligand **4**, *supplementary 1*, displayed a vibration peak at u 3241 cm^− 1^ belonging to u (NH), while the other u (NH) appeared at u 3118 cm^− 1 and was^ assigned to the intramolecular hydrogen bond of the form [N’-H…O = C] and the possibility of *trans-cis isomerization* of benzoyl thiourea, which affects the vibrational positions and properties of the central [-C(O)NHC(S)N’H-] moiety. The intramolecular hydrogen bond between (C = O) and thiourea forms a six-membered ring that can stabilize the molecular structure of ligand **4**. The lack of u (SH) at approximately 2500–2600 cm^− 1^ indicated the absence of the u (N = C-SH) tautomeric form, and ligand **4** remained in the form of thioketoamine. Absorption bands at u 1155 cm^− 1^, u 1331 cm^− 1^ and u 940 cm^− 1^ are assigned to u_asym_(N-CN’) thioamide band II, u_sym_ (N-CN’) thioamide band III and thioamide band IV, respectively. The vibration of u (C = O) at 1672 cm^− 1^ appeared lower than its ordinary absorption (∼1700 cm^− 1^), which was attributed to both intramolecular hydrogen bonds and the conjugation interchange between C = O and the aromatic ring [34–36]. The FT-IR spectra of copper and cadmium complexes **5 and 6**, *supplementary 2 and 3, respectively*, demonstrate a vibration band of u (C = S) at 1256 cm^− 1,^ which is relatively lower than that of ligand **4**, suggesting the coordination of the sulfur thioamide group. The coordination of the (S) shape conformation of the thiourea derivative with metal ions causes ligand-metal charge transfer, which decreases the double bond character of the C = S bond and permits a lower frequency shift of the thioimide band (IV). The change in (O-H) and u (N-H) vibrations compared to ligand **4** indicated their participation in coordination. In all copper complexes, the value of u (C = O) remains unchanged or is shifted, signifying its nonbonding nature. In the PVC spectrum, the characteristic absorption bands can be found in *supplementary 4* at 692 cm^− 1^ due to C-Cl stretching vibration, at 750, 922, 1063 and 1189 cm^− 1^, revealing the PVC chain, at 1348 and 1437 cm^− 1^, due to the C-H_2_ bend, at 2888 due to C-H_2_ stretching and at 2949 cm^− 1^ corresponding to C-H stretching, neighboring CH-Cl groups [54, 55]. The IR spectrum of the PVC/**4**, *supplementary 5*, composite exhibited the characteristic vibration of the PVC at u 2910, 2727, 2365, 1434, 1199, 921, 695 cm^− 1^, respectively. The vibration bands corresponding to ligand **4** in the composite appeared at 3655 ^cm−1^ (N-H), 3427 cm^− 1^ u (N’-H), 1685 cm^− 1^ u (C = O), 1329 cm^− 1^ u_asym_ (N-CN’) thioamide band III, 1252 cm^− 1^ u_asym_ (N-CN’) thioamide band II, and 839 cm^− 1^ u_asym_ (N-CN’) thioamide band IV. The IR spectra of the metal-containing composites PVC/**5** and PVC/**6**, *supplementary 6 and 7, respectively*, exhibited the characteristic vibration of PVC at 2921, 2863, 2727, 1432, 1331, 1194, 912, 702 cm^− 1^, respectively. The vibration bands correspond to complexes **5** and **6** in their appropriate compounds appearing at 3369 cm^− 1^ u (N-H), 3424, 3427 cm^− 1^ (N-H), 1679 cm^− 1^ u (C = O), 1331 cm^− 1^ u_asym_ (N-CN’) thioamide band III, 1194 cm^− 1^ (N-CN)_asym_ thioamide band II, and 840 cm^− 1^ (N-CN)_asym_ thioamide band IV.

### UV absorption

The bandgap energy is calculated from the equation ΔE = hc/λ, where ΔE is the bandgap energy (eV), h = 6.625 × 10^− 34^ JS, c = 3 × 10^8^ m/s, and λ is the wavelength. The electronic spectrum of ligand **4** showed absorption bands at λ 225 nm, λ 276 nm, and λ 322 nm, and their bandgap energies were 5.51 eV, 4.49 eV, and 3.85 eV, respectively. The absorption peaks at λ 225 and 276 nm are due to the π–π* transition of the aromatic rings. The absorption peak at λ 322 nm is due to the n-π* transition. Copper complex **5** exhibited absorption bands at λ 276 nm and λ 321 nm, and their bandgap energies were 4.49 eV and 3.86 eV, respectively. Cadmium complex **6** showed bands at λ 275 nm and λ 320 nm, and their bandgap energies were 4.51 eV and 3.87 eV, respectively. Roughly weighed amounts (4–6 mg) of PVC composites were dissolved in THF (3.0 mL). A volume of 0.1 ml of the solution was withdrawn, diluted to 3.0 ml and analysed by UV‒vis spectrophotometry. The electronic absorption spectrum of the PVC film displayed one characteristic band at λ 255 nm, and its bandgap energy was 4.86 eV, while the PVC/**4** composite showed absorption bands at λ 260 nm, λ 265 nm, and λ 302 nm, and their bandgap energies were 4.77 eV, 4.68 eV, and 4.11 eV, respectively. On the other hand, metal-containing composites PVC/**5** and PVC/**6** showed absorption bands at λ 260 nm, λ 265 nm, and λ 300 nm, and their bandgap energies were 4.77 eV, 4.68 eV, and 4.13 eV, respectively.

### NMR spectra

*The*^1^H-NMR spectrum of free ligand **4** in DMSO-d6, *Supplementary 8*, displayed two singlet signals at δ 12.54 ppm and δ 11.59 ppm assigned to the protons of NH- (thioamide) and NH- (amide), respectively. Aromatic protons exhibited a doublet signal at δ 7.97 ppm (*J* 8 Hz) assigned to (*H*-C3, *H*-C5, *H*-C3’, *H*-C5’) protons, a multiplet signal at δ 7.66 ppm assigned to (*H*-C11, *H*-C12, *H*-C13, *H*-C11’, *H*-C12’, *H*-C13’) protons, two doublet signals at δ 7.53 ppm (*J* 8 Hz, *J* 7.5 Hz) corresponds to (H-C10, H-C14, H-C10’, H-C14’) protons, and a doublet signal at 7.09 ppm (*J* 8.5 Hz) assigned to (H-C2, H-C6, H-C2’, H-C6’), respectively. The low field positions of most signals are evidence of various H-bonding interactions, such as intramolecular NH–O = C-, intermolecular DMSO⋯HN-C = S, or intermolecular hydrogen bonding between ligand molecules [56]. It is noteworthy that the most likely opposite orientation between the C = S and C = O bonds favored the highly stable S-conformation in such thiourea derivatives more than the M- or U- geometries. The C = O and H-N groups in the S shape conformation form a pseudosix-membered ring through an intramolecular hydrogen bond [57]. The ^13^ C-NMR spectrum of ligand **4** in DMSO-d6 (*Supplementary 9*) showed two signals at δ 179.24 ppm and 168.25 ppm assigned to thioamide and amide carbons, respectively. The aromatic carbons resonated at δ 154.52 (C-1, C-1’), δ 133.58 (C-9, C-9’), δ 133.14 (C-4, C-4’), δ 132.15 (C-11, C-11’), δ 128.69 (C-13, C-13’), δ 128.58 (C-10, C-10’), δ 128.45 (C-14, C-14’), δ 128.29 (C-3, C-3’), δ 126.35 (C-5, C-5’), δ 121.76 (C-2), δ 119.75 (C-2’), δ 118.68 (C-7), and δ 117.89 (C-7’). Interestingly, the spectral data of complexes **5** and **6** proved the formation of one stable structure and that the NH protons have not been lost through thioenolization of the C = S group; thus, the sulfur atom coordinates to the metal atoms. The ^1^ H-NMR spectrum of copper complex **5** (*Supplementary 10*) displayed shielded singlet signals of the thioamide and amide NH protons at 10.95 ppm and 10.85 ppm, while all other aromatic protons resonated as three multiplet signals at δ 8.91–7.80, δ 7.66–7.53 and δ 7.20–6.85. Interestingly, the thioamide proton signal in copper complex **5** is shifted upfield relative to its parent ligand **4**. This shift revealed the participation of the thioamide in bonding to the copper ion and was thus consistent with the IR data. The ^13^ C-NMR spectrum of complex **5** in DMSO-d6, (*Supplementary 11*), showed two signals at δ 179.24 ppm and δ 168.25 ppm assigned to thioamide and amide carbons, respectively. Other aromatic carbons resonated at δ 154.52 ppm (C-1, C-1’), δ 133.58 ppm (C-9, C-9’), δ 133.14 ppm (C-4, C-4’), δ 132.78 ppm (C-11, C-11’), δ 129.19 ppm (C-13, C-13’), δ 128.50 ppm (C-10, C-10’), δ 128.22 ppm (C-14, C-14’), δ 120.79 ppm (C-3, C-3’), δ 120.76 ppm (C-5, C-5’), δ 120.73 ppm (C-2) and δ 120.68 ppm. The ^1^ H-NMR spectrum of cadmium complex **6** (*Supplementary 12*) exhibited two singlet signals at 12.56 ppm and 11.52 ppm corresponding to the protons of thioamide and amide NH, respectively, while other aromatic protons resonated at δ 7.99 ppm (d, 4 H, *J* 7.2 Hz, H-C3, H-C5, H-C3’, H-C5’), δ 7.93 ppm (m, 6 H, H-C11, H-C12, H-C13, H-C11’, H-C12’, H-C13’), δ 7.68 ppm (2d, 4 H, *J* 8 Hz, 7.5 Hz, H-C10, H-C14, H-C10’, H-C14’), δ 7.36 ppm (d, 4 H, *J* 8.5 Hz, H-C2, H-C6, H-C2’, H-C6’). The ^13^ C-NMR spectrum of complex **6** in DMSO-d6, (*Supplementary 13*), showed two signals at δ 179.19 ppm and δ 168.21 ppm assigned to the thioamide and amide carbons, respectively. Other aromatic carbons were at δ 154.48 (C-1, C-1’), δ 133.55 (C-9, C-9’), δ 133.09 (C-4, C-4’), δ 132.78, δ 132.09 (C-11, C-11’), δ 129.20, δ 128.61 (C-13, C-13’), δ 128.49 (C-10, C-10’), δ 128.40 (C-14, C-14’), δ 126.24 (C-5, C-5’), δ 119.55 (C-2’), δ 118.87 (C-7), and δ 118.63 (C-7’).

### Surface morphology studies

**Fig. 1:**
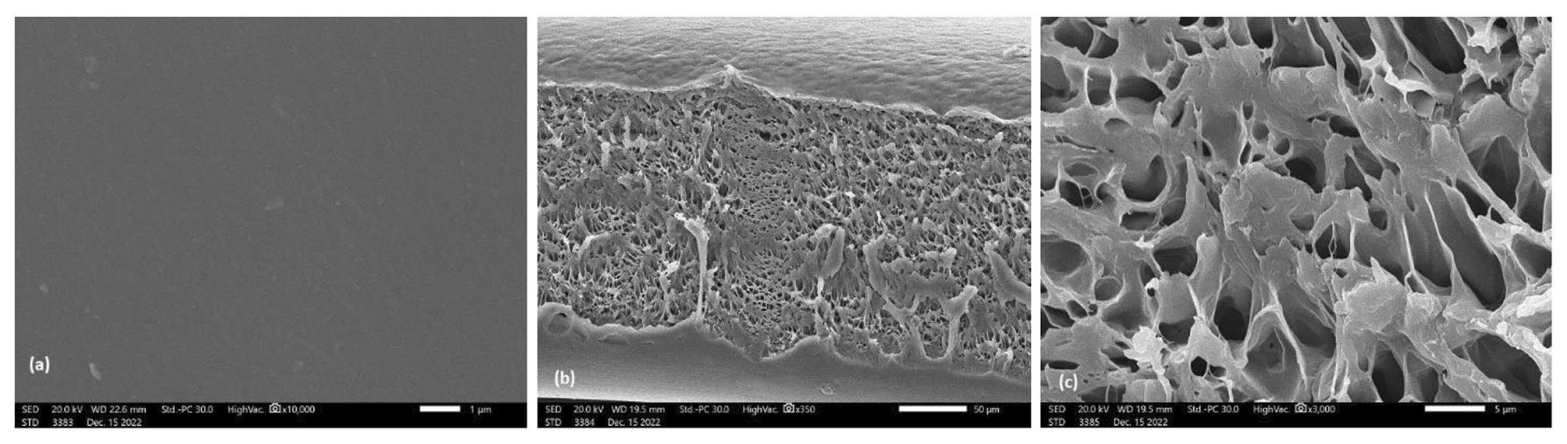
SEM images of (a): PVC film; (b) and (c) cross sections [scale bar 50 mm and 5 mm, respectively]

**Fig. 2:**
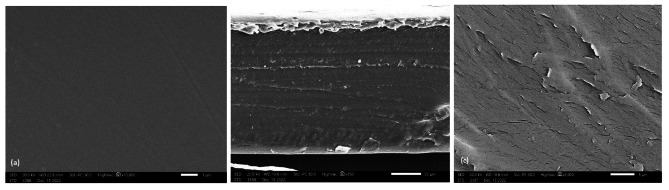
SEM image of (a): PVC/**4** film; (b) and (c) cross sections [scale bar 50 μm and 5 μm, respectively]

**Fig. 3:**
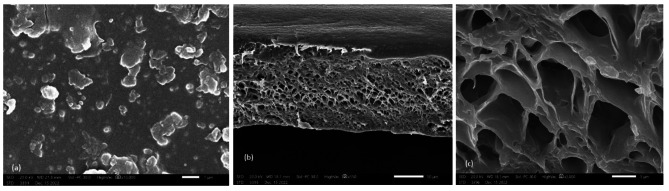
SEM image of (a): PVC/**5** film; (b) and (c) cross sections [scale bar 50 μm and 5 μm, respectively]

**Fig. 4:**
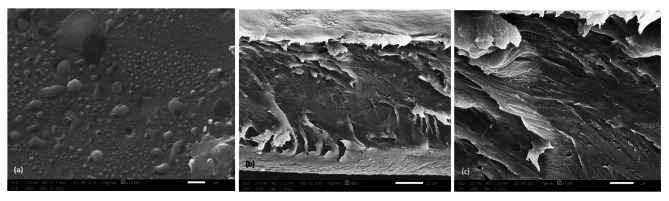
SEM image of (a): PVC/**6** film; (b) and (c) cross sections [scale bar 20 μm and 5 μm, respectively]

The SEM images proved that the morphologies of the materials were highly influenced by the nature of the composite incorporated into the matrix. The control PVC film (Fig. 1_a_) can be seen as the most transparent and smooth topography and consequently is the most homogeneous. The cross section [Fig. 1_b_ and 1_c_ (50 mm and 5 mm width, respectively)] assumed that the PVC surface is rough and has a regular porous structure in addition to many distributed craters equipped with 1–2 μm cavities. The SEM image of the PVC film containing ligand **4**, Fig. 2_a_, shows that the morphological feature of the PVC surface at a magnification power of 1 mm is smooth, neat and has no cracks with some flaws. The cross section (Fig. 2_b_, 2_c_) at a magnification power of 50 mm and width of 5 mm showed that the nonporous PVC surface contained cracks. The obtained nonporous PVC composite structure is attributed to the increase in solution intrinsic viscosity [58]. The SEM image of the PVC film containing composite **5** (Fig. 3_a_) showed a rough surface, and the incorporated antimicrobial agent was irregularly distributed, causing damage within the PVC surface homogeneity and the formation of several cracks. Cross-section images (Fig. 3_b_ and 3_c_) at magnification powers of 50 mm and 5 mm width, respectively, showed a rough-porous structure containing many craters equipped with 1–2 μm cavities. The SEM image of the PVC film containing composite **6** (Fig. 4_a_) showed a rough surface, and the incorporated antimicrobial agent was irregularly distributed, causing damage to the PVC surface homogeneity. The cross sections (Fig. 4_b_ and 4_c_) at magnification powers of 50 mm and 5 mm, respectively, showed that the nonporous PVC surface contained cracks.

## Thermal analysis

**Fig. 5:**
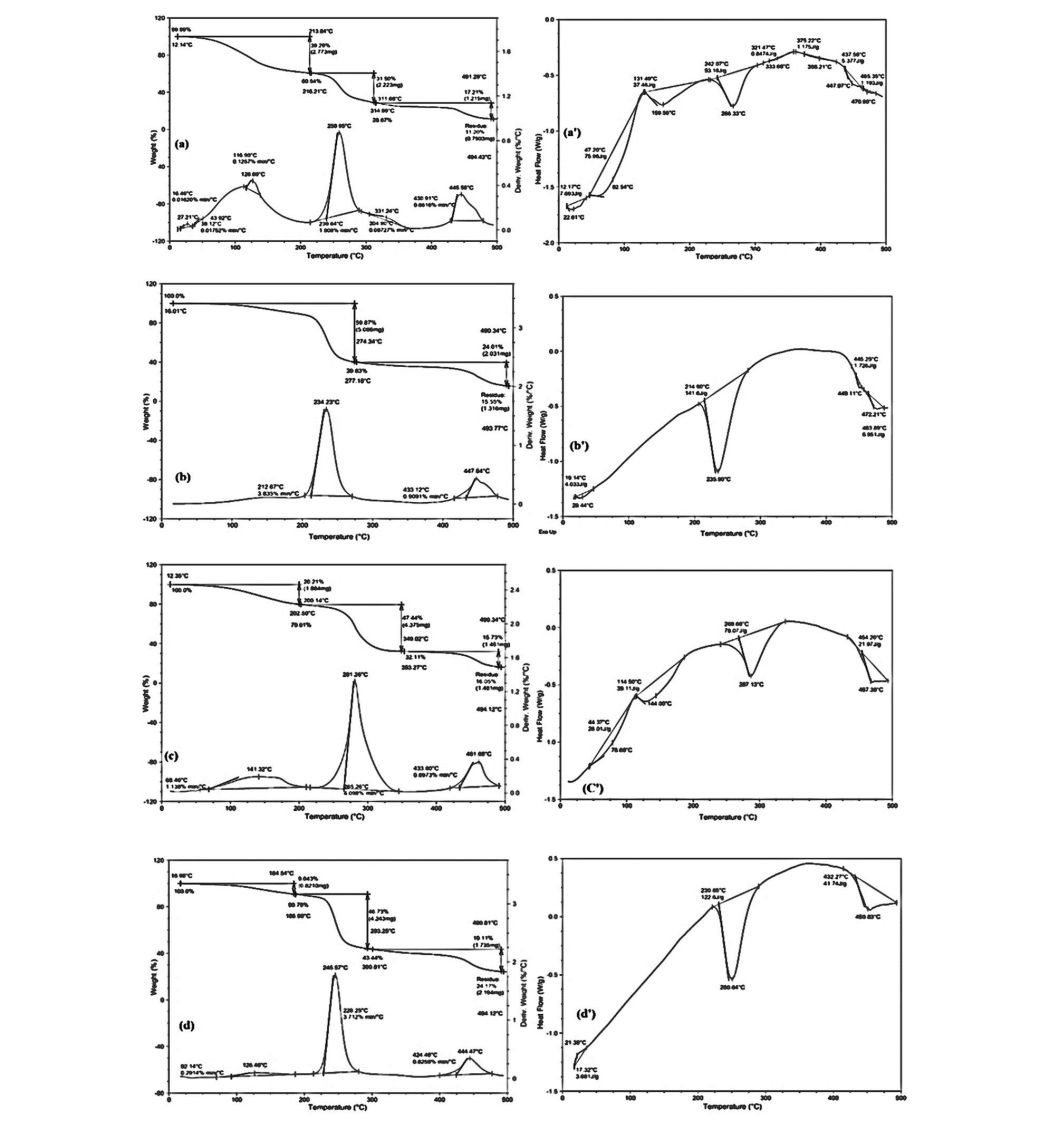
TGA/DTG and DSC curves of: (a,a’) PVC; (b,b’) PVC/**4**; (c,c’) PVC/**5** and (d,d’) PVC/**6**

Thermal stability is characterized by the weight loss of the sample after heating in the temperature range of 25–500 °C. Thermal data of ligand **4** and its metal complexes **5** and **6** were examined utilizing thermogravimetric analysis (TGA), derivative thermogravimetric analysis (DrTGA) and differential thermal analysis (DTA) methods under a nitrogen atmosphere. The TGA/DrTGA profile curves (*Supplementary 14*) of ligand **4** displayed successive decomposition in four cumulative stages at 290.1 °C, 400 °C, 500 °C, 700 °C, leaving at >700 °C a 14.12% value of the sample weight as a residue. The DTG showed a strong main endotherm at 227.57 °C and two other weak endotherms at 44.16 and 407.18 °C. Thermal studies of complexes **5** and **6** proved their unambiguous thermal stability. Heating the samples of both complexes up to 700 °C left 33% and 24% of the sample weights, respectively, as remaining metal oxide residues [59]. The TGA/DrTGA profile curves (*Supplementary 15*) of copper complex **5** exhibited decomposition steps at 153.48 °C (-2.18%, wt loss), 279.84 °C (-34.49%, wt loss), 458 °C (-21.05%, wt loss), 526 °C (-2.29%, wt loss), 597 °C (-3.08%, wt loss), and 700 °C (-4.19%, wt loss). The DTG showed a strong main endotherm at 231.86 °C, one week endotherm at 132.18 °C and two weak broad endotherms at 510.24 and 550.94 °C. The TGA/DrTGA profile curves (*Supplementary 16*) of copper complex **6** exhibited decomposition steps at 107.90 °C (-1.72%, wt loss), 260.44 °C (-28.08%, wt loss), 402 °C (-24.87%, wt loss), and 699 °C (-20.96%, wt loss). The DTG curve analysis curve exhibited a weak endotherm at 91.02 °C, a strong main endotherm at 227.34 °C, and a broad endotherm at 338.18 °C. As illustrated in Fig. 5, the thermal decomposition of all PVC-based films occurs through three degradation steps in the temperature range 213–445 °C, attributed to dehydrochlorination and degradation of the dehydrochlorinated residuals of PVC film samples [60]. At temperatures higher than 213 °C, the chain radical mechanism becomes relevant, and the melting of C-C or C-H bonds takes place [61]. The maximum degradations of the PVC film sample were at 213 °C, 259 and 445 °C with 39%, 31% and 17% weight losses, respectively. The first degradation peak at approximately 116 °C was ascribed to the loss of contaminated moist THF. The residue was 11% of its weight. The maximum degradation temperatures of the PVC/ligand **4** film were 212 °C, 234 and 447 °C with 3.8%, 60% and 24% weight losses, respectively. Interestingly, the degradation peaks at approximately 212 and 445 °C exhibited lower mass losses than that of the neat PVC sample. However, the mean degradation temperature was lower than that of the neat sample, and the degradation process exhibited a higher mass loss. This result showed an improvement in thermal resistance that was associated with incorporation of the ligand within the polymeric chain. The thermal degradation temperatures of PVC-based films incorporating copper composite **5** were 202 °C, 353 °C, and 462 °C with 20%, 47% and 16%, respectively, mass losses, leaving 16% of its weight as a CuO residue. Interestingly, the thermal degradation temperatures of PVC-based films incorporating cadmium composite **6** were 187 °C, 293 °C, and 444 °C with 9%, 47% and 19%, respectively, mass losses, leaving 24% of its weight as a CdO residue. The observed shifted thermal degradations can be attributed to the presence of organometallic nanoparticles that strengthened van der Waals interactions between the PVC chains and therefore improved the thermal stability of the film [62].

### Antimicrobial activity

The antimicrobial activity of organic ligand **4** and its Cu(I) **5** and Cd(II) **6** complexes were tested against strains of gram-positive bacteria (*S. pneumoniae RCMB 010010 and B. subtilis RCMB 010067*), gram-negative bacteria (*E. coli RCMB 010052* and *P. aeruginosa RCMB 010043*) and pathogenic fungi (*C. albicans RCMB 05036* and *(A) fumigatus RCMB 02568*) using DMSO as a negative control. Gentamicin, ampicillin, and amphotericin B were used as antimicrobial and antifungal positive controls, and the results are summarized in Table 1. The average diameter of the bacterial inhibition zone was correlated with the antibacterial activity of the corresponding substrate, that is, the larger the clear area around the well, the higher the inhibitory efficiency. Compared to the standard gentamicin, all tested substrates **4**–**6** exhibited high activity against gram-negative bacteria, particularly Cd(II) complex **6**. The order of their activities was **6** > **5** > **4**. However, all substrates tested (**4**–**6)** showed moderate to good activity against gram-positive bacteria compared to standard antibiotics. The order of their activities against *S. pneumoniae RCMB 010010* was **6** > **4** > **5** and *against (B) subtilis RCMB 010067* was **6** > **5** > **4**. Similarly, the investigated substrates showed high activity against pathogenic fungi (*C. albicans RCMB 05036* and *A. fumigatus RCMB 02568*), and their activity order was **6** > **5** > **4**.

**Table 1:** Results of the antimicrobial screening of ligand **4** and its metal complexes **5, 6**

Compound	Tested Microorganisms					
	(G ram negative Bacteria)		(Gram positive Bacteria)		(Fungi)	
	E.Coli	P.Aeruginosa	S. Pneumoniae	B. subtilis	C. albicans	A. fumigatus
	Inhibition zone diameter (mm/mg sample)					
Gentamicin(G-)	19.9 ± 0.3	17.3 ± 0.1	-------	-------	-------	-------
Ampicillin (G+)	-------	-------	23.8 ± 0.2	32.4 ± 0.3	-------	-------
Amphotericin B	-------	-------	-------	-------	25.4 ± 0.1	23.7 ± 0.1
Control: DMSO	NA	NA	NA	NA	NA	NA
**4**	15.1 ± 0.25	13.2 ± 0.58	18.1 ± 0.25	16.3 ± 0.58	16.2 ± 0.25	14.3 ± 0.58
**5**	16.2 ± 0.25	13.9 ± 0.58	17.2 ± 0.63	18.6 ± 0.63	17.1 ± 0.25	15.2 ± 0.58
**6**	19.2 ± 0.63	16.2 ± 0.58	18.3 ± 0.58	19.6 ± 0.44	21.3 ± 0.72	20.6 ± 1.2

NA = absence of activity; the data are expressed in the form of mean ± standard deviation

**Fig. 6:**
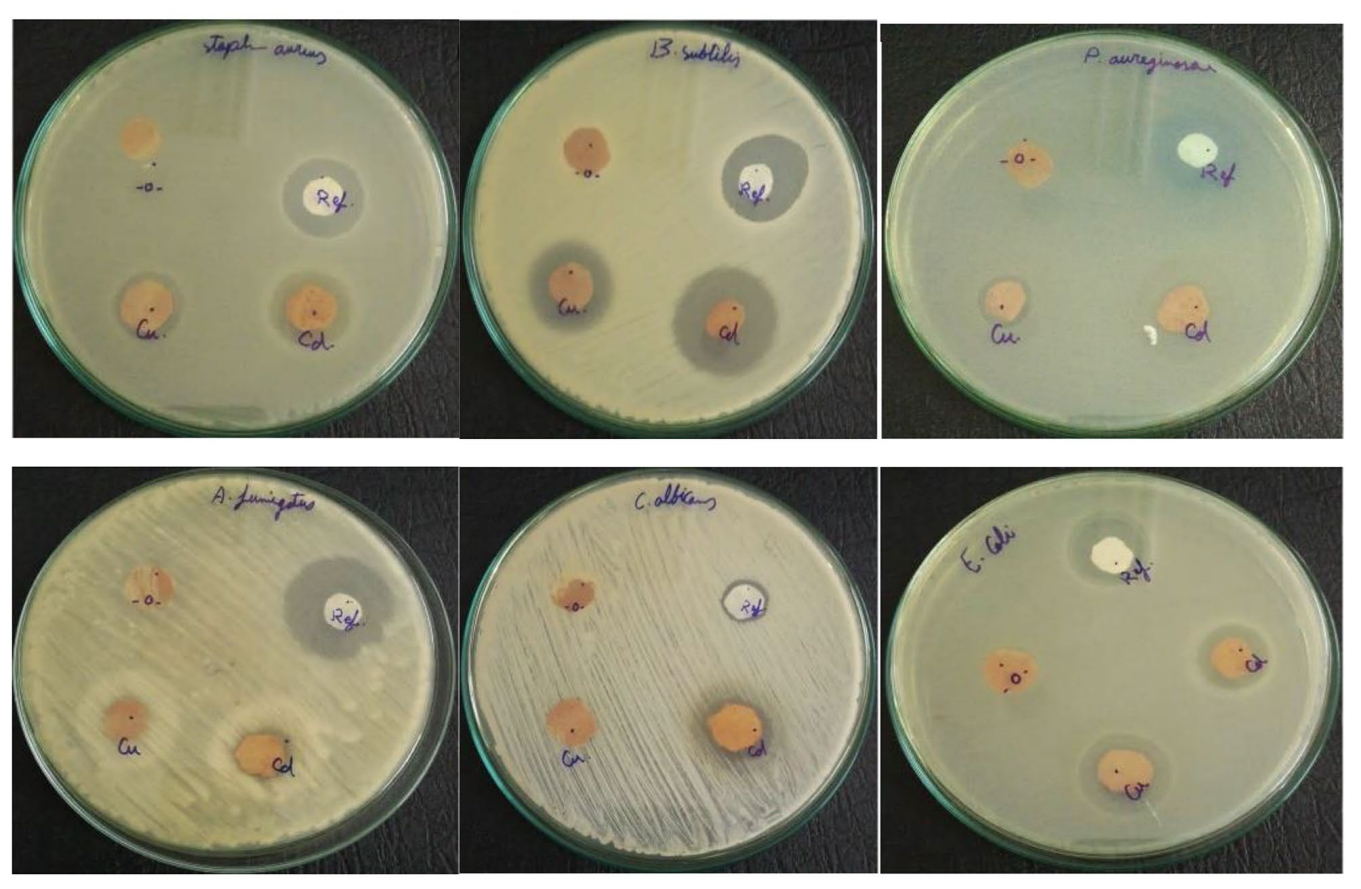
Photographic images of microbial inhibition zones against *S. Pneumoniae, B. subtilis, E. coli, P. aeruginosa, C. albicans, and A. fumigatus*. Key: PVC (Ref), PVC/**4** (-O-), PVC/**5** (Cu), PVC/**6** (Cd)]

The antimicrobial activity of the PVC, PVC/**4**, PVC/**5**, and PVC/**6** composite films against *S. aureus ATCC 25,923, B. subtilis RCMB 015 (1) NRRL B-543, E. coli ATCC 25,922, P. aeruginosa ATCC 27,853, C. albicans RCMB 005003 (1) ATCC 10,231*, and *(A) fumigatus RCMB 002008* are compiled in Table 2, and photographic images of microbial inhibition zones are shown in Fig. 6. Broad-spectrum gentamicin and ketoconazole B were used as antimicrobial and antifungal positive controls, respectively. The PVC film exhibited inhibitory activity toward the microorganisms studied. The inhibitory zones for the PVC film presented superior activity for *P. aeruginosa ATCC 27,853, (B) subtilis RCMB 015 (1) NRRL B-543* and *A. fumigatus RCMB 002008*, with average diameters of halos equal to 29 ± 0.25 mm, 30 ± 0.14 mm and 35 ± 0.22 mm, respectively. Compared to standard antibiotics, the inhibitory zones of the PVC film presented good activity against *E. coli ATCC 25,922, S. aureus ATCC 25,923*, and *(C) albicans RCMB 005003*, with average halo diameters equal to 27 ± 0.31 mm, 27 ± 0.25 mm and 17 ± 0.20 mm, respectively.

**Table 2:** Antimicrobial screening results of PVC and PVC/**4-**PVC/**6** composites films

Compound	Tested Microorganisms					
	(G ram negative Bacteria)		(Gram positive Bacteria)		(Fungi)	
	*E.Coli*	*P.Aeruginosa*	*S. aureus*	*B. subtilis*	*C. albicans*	*A. fumigatus*
	Inhibition zone diameter (mm/mg sample)					
Gentamicin(G-)	30 ± 0.25	29 ± 0.30	24 ± 0.22	26 ± 0.32	--------	--------
Ketoconazole	-------	-------	-------	-------	20 ± 0.23	17 ± 0.29
Control: DMSO	NA	NA	NA	NA	NA	NA
PVC	27 ± 0.31	29 ± 0.25	27 ± 0.25	30 ± 0.14	17 ± 0.20	35 ± 0.22
PVC/**4**	NA	NA	NA	NA	NA	NA
PVC/**5**	29 ± 0.33	17 ± 0.36	21 ± 0.20	22 ± 0.30	NA	NA
PVC/**6**	24 ± 0.27	16 ± 0.11	22 ± 0.22	32 ± 0.25	22 ± 0.35	NA

NA = absence of activity; the data are expressed in the form of mean ± standard deviation

It is important to note that the active film containing the ligand PVC/**4** did not show antimicrobial action against the microorganisms studied, as the formation of the inhibition zone was not observed. One conceivable reason for the absence of activity may be due to the expected formation of a hydrogen-bonded complex network structure that prevents the connection between the bacterial cells and the polymer surface active functionality that confirms the initial adhesion of the bacterial cells. The resistance mechanisms of bacterial species to antibiotics and disinfectants are caused by their intrinsic resistance due to the decreased permeability of the cell wall, acquired resistance produced by many mutations in chromosomal genes, acquisition of resistance genes by horizontal transfer facilitated by bacteriophages, transposons, plasmids, and growth in biofilms that contribute to the progress of phenotypic resistance [2]. The PVC/copper composite PVC/**5** did not produce any inhibition zones for *C. albicans RCMB 05036* and *A. fumigatus RCMB 02568*, indicating no antifungal activity. Interestingly, the PVC/cadmium compound PVC/**6** did not show inhibition zones for *A. fumigatus RCMB 02568*, while its inhibition zones for *C. albicans RCMB 05036* with an average halo diameter equal to 22 ± 0.35 mm indicated excellent antifungal activity. It should be noted that C. albicans is the most common fungus involved in fungal infections in humans. It is an opportunistic pathogen that is a component of the normal microbiota of the oropharynx, gastrointestinal tract, skin, vagina and oral cavities. It can colonize several biomedical devices made of metal and silicon and stay in the body, causing recurrent infections [63]. On the other hand, *P. aeruginosa* is the most resistant bacterial species to disinfectants and antibiotics, whereas *S. aureus* is the foremost pathogen of community-acquired infections and nosocomial infections, and the cell wall of the latter is nearly 10 times thicker than that of the former [64]. From this study, the antibacterial power of PVC/cadmium composite PVC/**6** is significantly higher against the mentioned bacterial strains compared to PVC/copper composite PVC/**5**. Therefore, these results are of great significance for research and should open an innovative direction in surface engineering with wide use of antimicrobial activity within the biomedical field [65].

## Conclusions

In this study, a new antimicrobial plastic material incorporating metal complexes of the oxybis (4,1) phenylene moiety flanked symmetrically by two phenylthioureas in a PVC film was successfully synthesized by a cheap and fast solvent casting technique. The bis acylthiourea derivative *N,N’- ((oxybis (4,1-phenylene))bis (azanediyl))bis (carbonothioyl))dibenzamide* and its Cu(I) and Cd(II) complexes were synthesized and characterized. The results revealed that on coordination, the thiourea derivative behaves as a neutral ligand, which coordinates the metal ion through the sulfur atom of the thiocarbonyl group. Analytical measurements clearly indicated the formation of the favored stable ligand-metal (S)-shaped conformation. The in vitro antimicrobial assessment achieved directly with various bacterial and fungal indicator strains showed excellent activities compared with standard antibiotics. Notably, the PVC/ligand film showed no antimicrobial action against all of the studied microorganisms, attributed to the hydrogen-bonded complex network structure of the expected active sites. The PVC/cadmium compound exhibited activity of 22 ± 0.35 mm against pathogenic *C. albicans RCMB 05036*, while its analog PVC/copper compound was inactive. The PVC/copper compound exhibited 29 ± 0.33% activity against pathogenic *E. coli ATCC 25,922* compared to 24 ± 0.27% activity of the PVC/cadmium analogue. The antibacterial power of the PVC/cadmium compound is significantly superior against the most resistant bacterial species to antibiotics and disinfectants, namely, *P. aeruginosa ATCC 27,853, S. aureus ATCC 25,923*, and *B. subtilis RCMB 015 (1) NRRL B-543*, compared to its PVC/copper composite analogue. Therefore, these results are of great significance for research and should open a new direction in surface engineering with antimicrobial activity for wide use within the biomedical field. Further challenges include the development of reusable antimicrobial polymers, a broad range of antimicrobial activity, and an activity-controlled system on demand sites.

## Electronic supplementary material

Below is the link to the electronic supplementary material.

Supplementary Material 1

## Data Availability

All data generated or analysed during this study are included in this published article [and its supplementary document].
