# Supplementary material for: A new antimicrobial PVC-based polymeric material incorporating bisacylthiourea complexes
Source: BMC Chem. 2023 May 3;17(1):44. doi: 10.1186/s13065-023-00958-7 (PMC10157947; doi:10.1186/s13065-023-00958-7)
Supplement: Supplementary file 1 — Supplementary Material 1 [file 13065_2023_958_MOESM1_ESM.pdf]

A New Antimicrobial PVC-based Polymeric Material Incorporating Bisacylthiourea Complexes

Hammed H. A. M. Hassan*, Amel F. ELhusseiny

Chemistry Department, Faculty of Science, Alexandria University, P.O. Box 426-Ibrahimia, 21321-Alexandria, Egypt. E-mail: hamed.hassan@alexu.edu.eg

Supplementary materials

Supplementary 1:

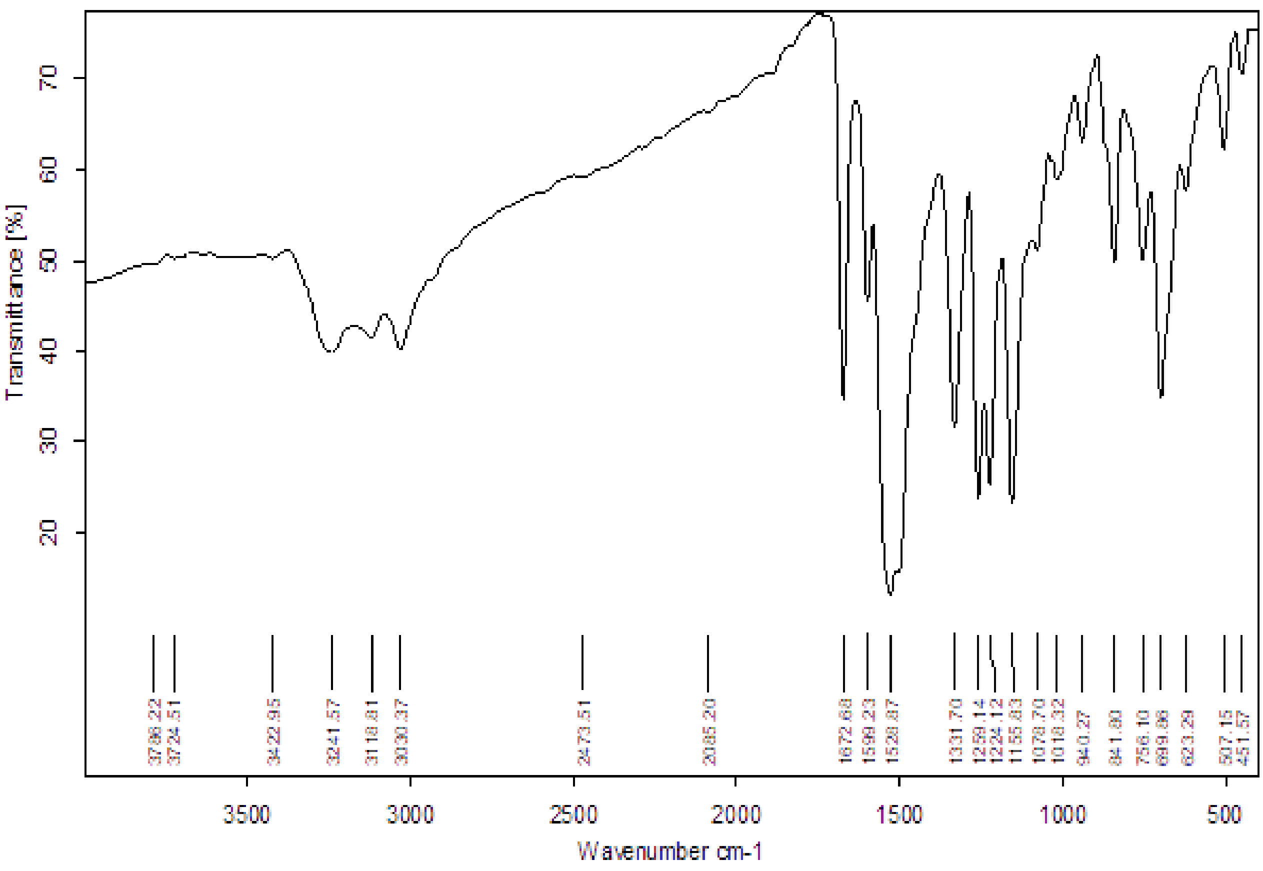

Figure S1:IR spectrum of N,N’-(((oxybis(4,1-phenylene))bis(azanediyl))bis(carbonothioyl))dibenzamide **4**

....

Supplementary 2

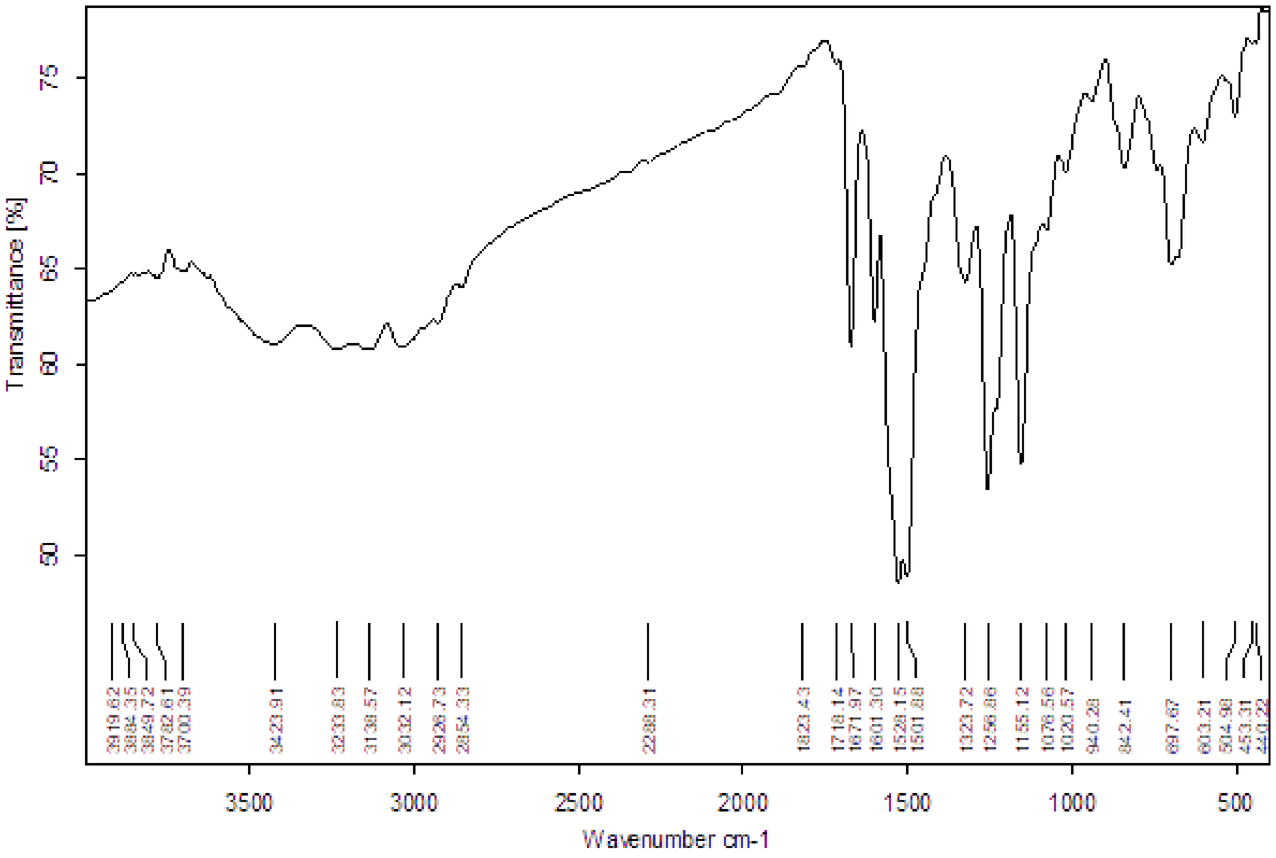

Figure S2: IR spectrum of the copper complex **5**

....

Supplementary 3

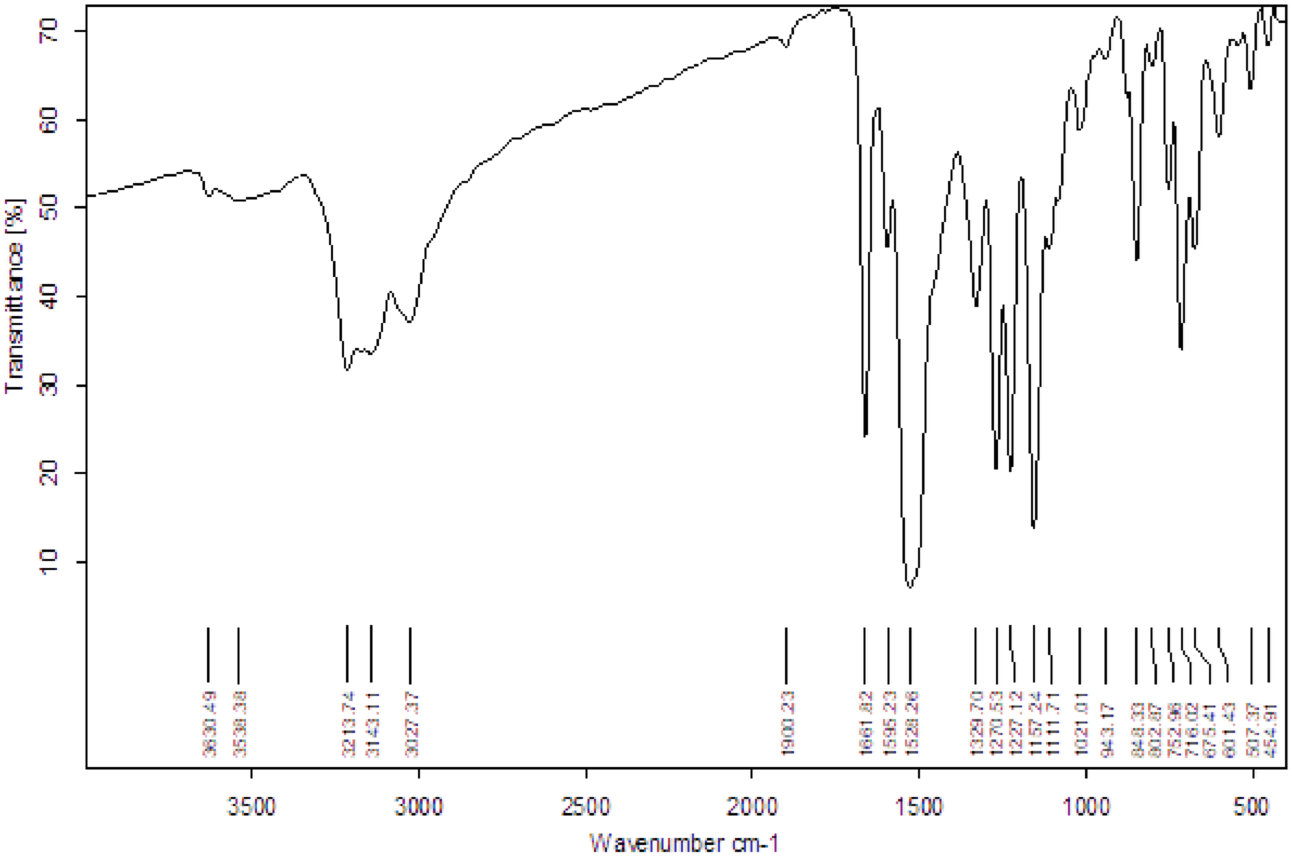

Figure S3: IR spectrum of the cadmium complex **6**

....

Supplementary 4

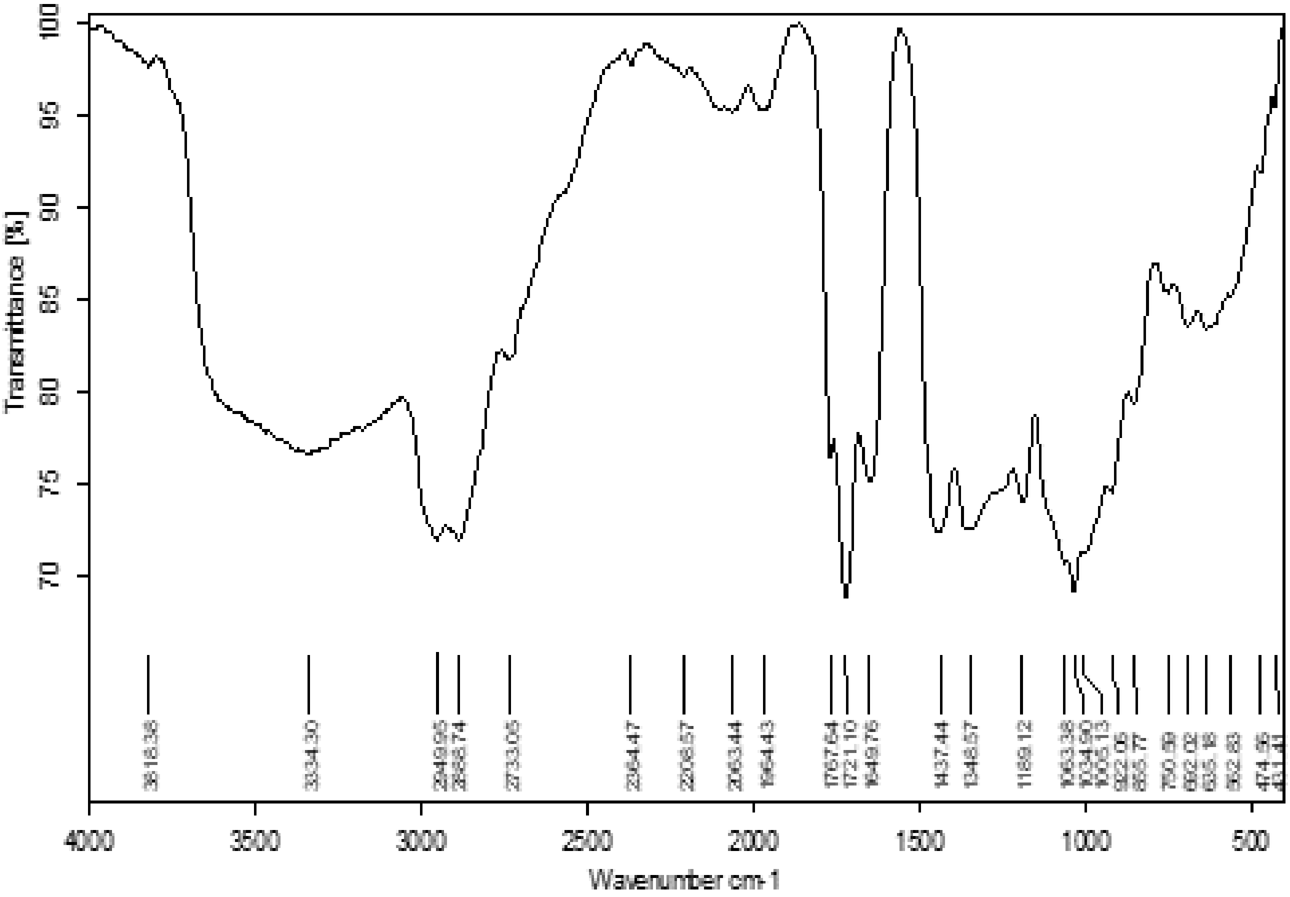

Figure S4: IR Spectrum of the prepared PVC film

……..

Supplementary 5

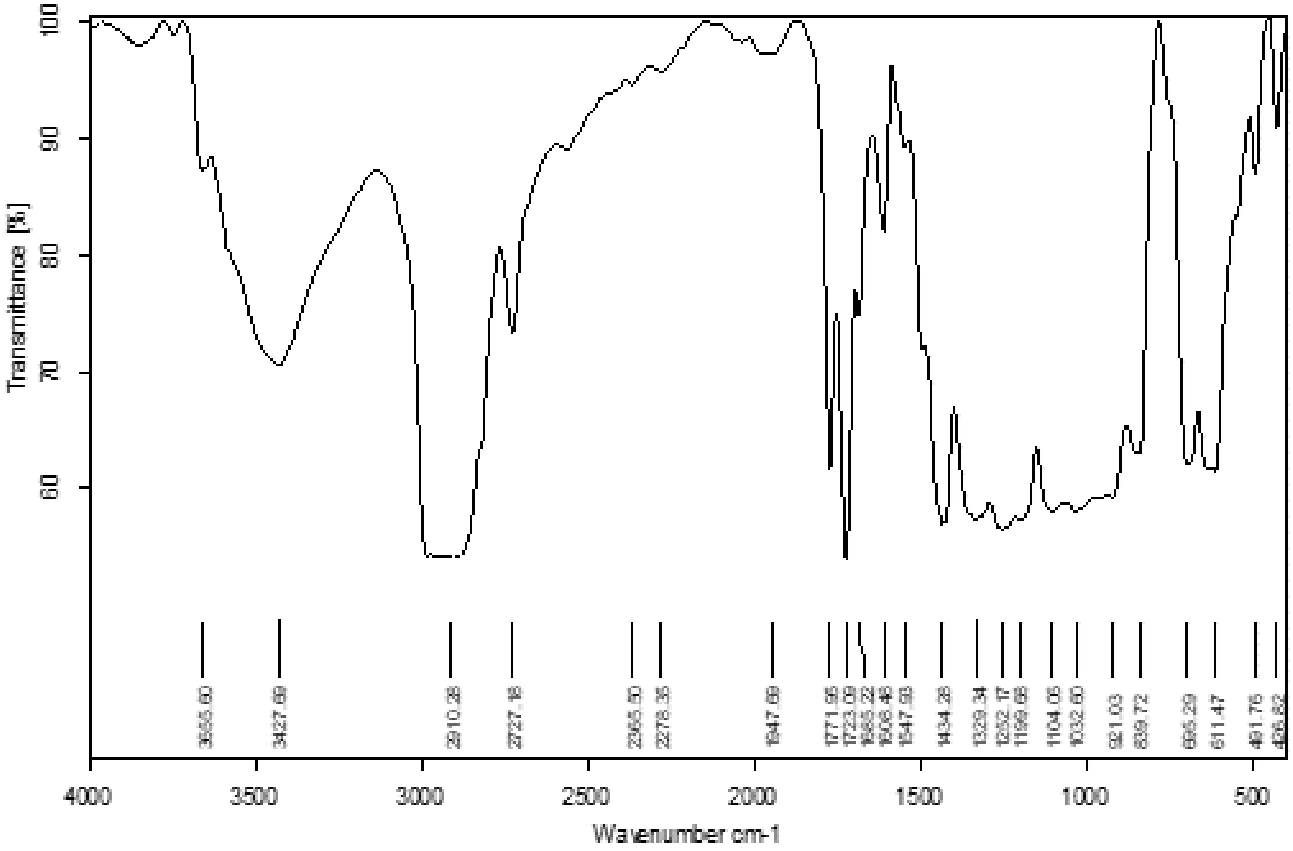

Figure S5: IR Spectrum of the prepared PVC/**4** composite film

…….

Supplementary 6

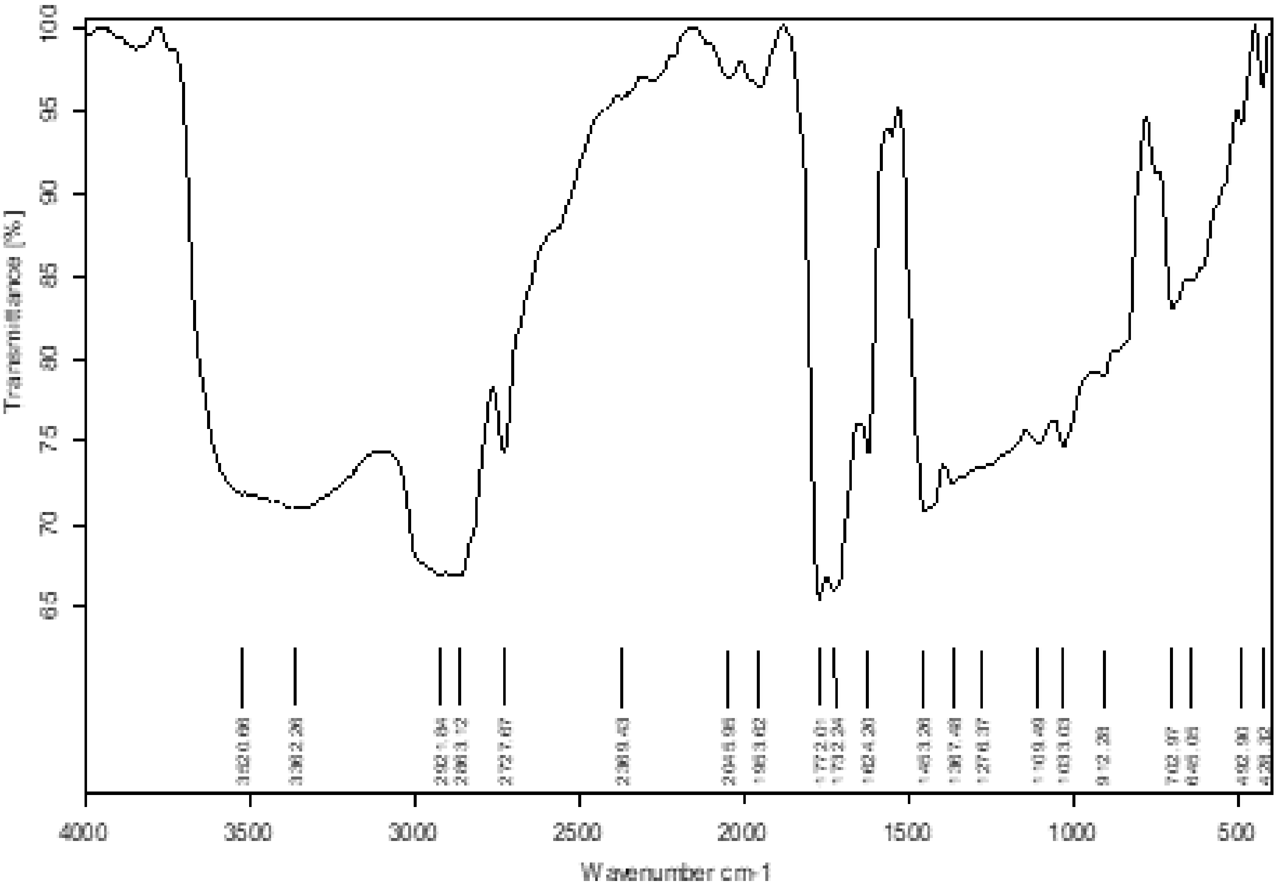

Figure S6: IR Spectrum of the prepared PVC/**5** composite film

....

(*Supplementary 7)*.

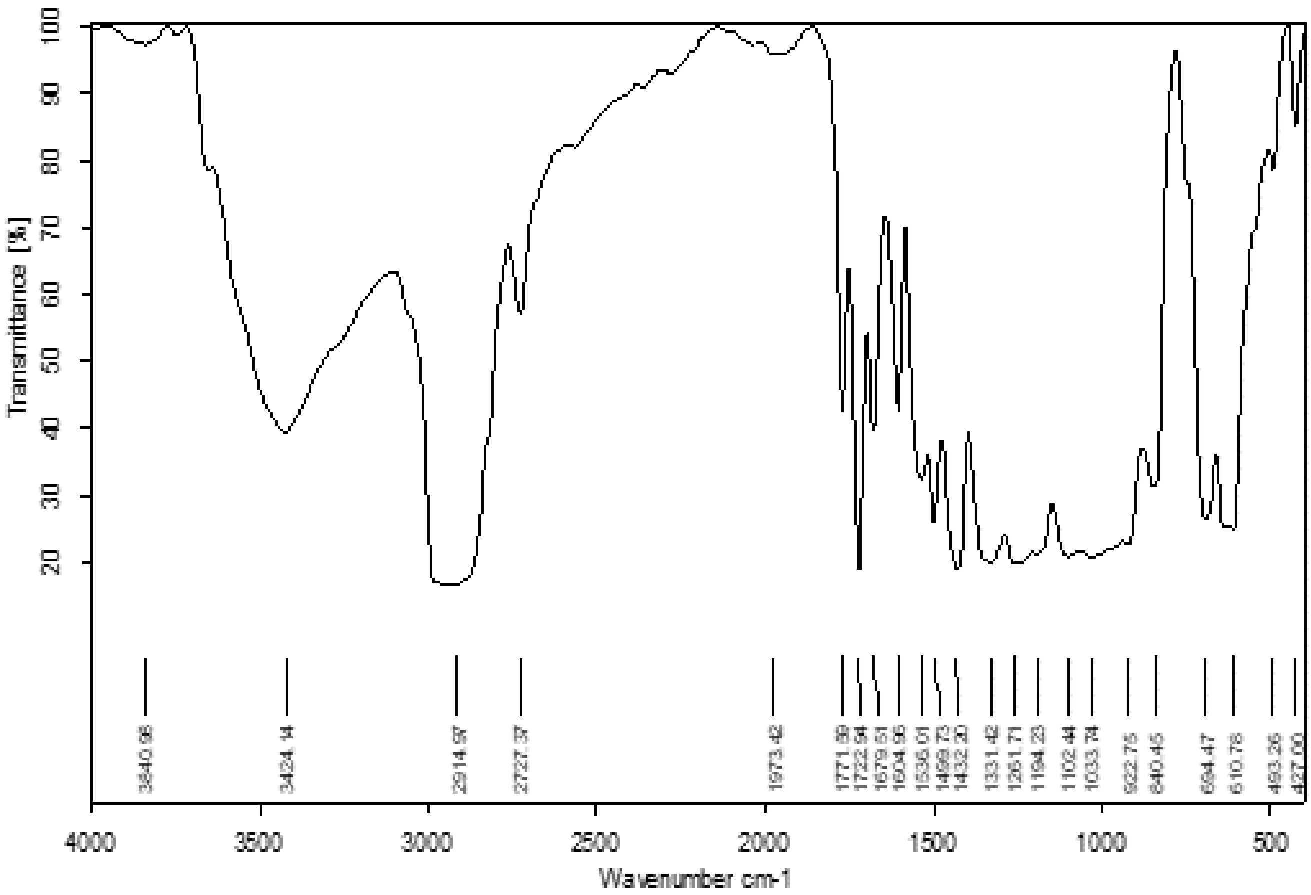

Figure S7: IR Spectrum of the prepared PVC/**6** composite film

……

(*Supplementary 8)*.

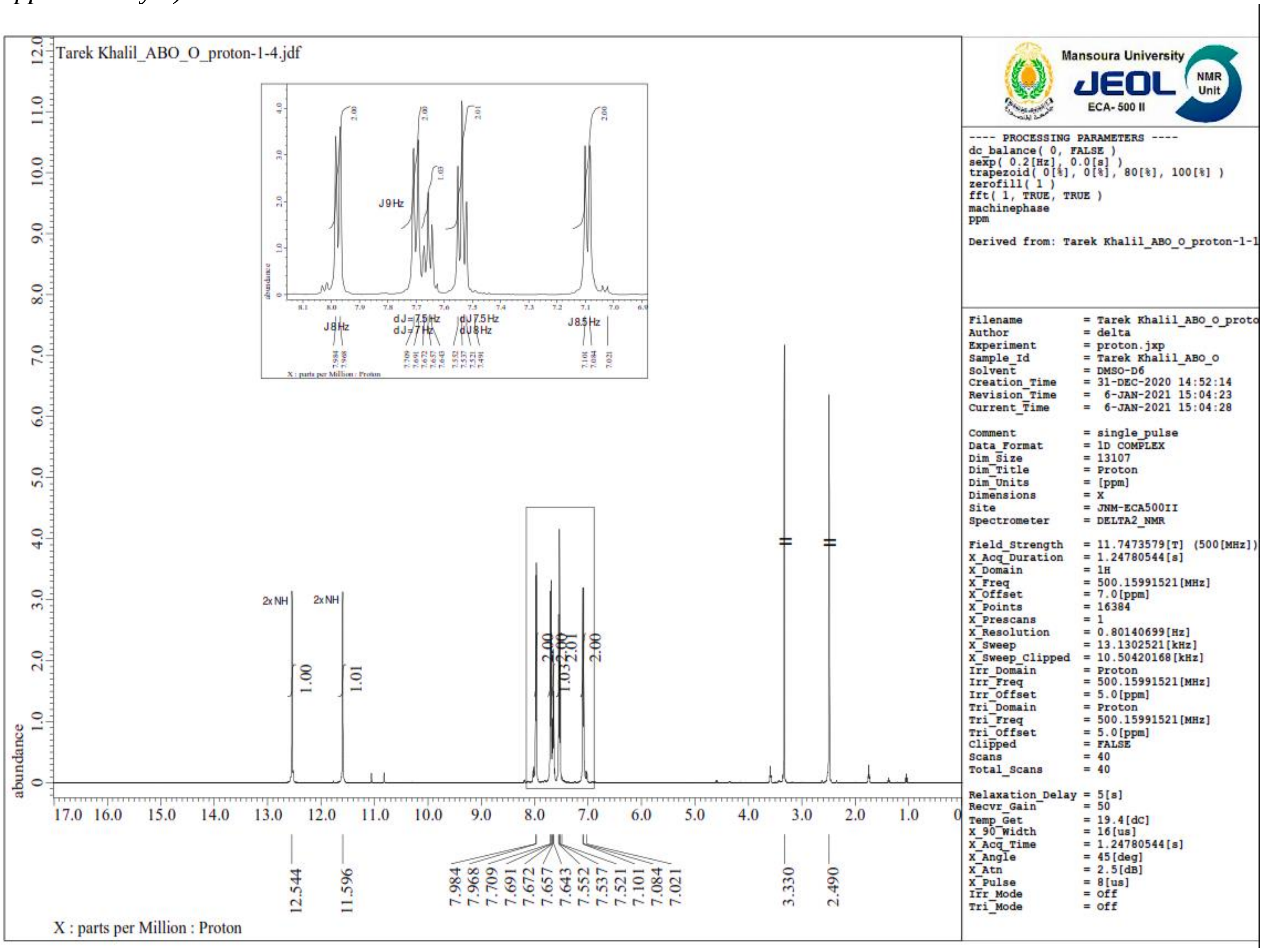

Figure S8: ^{1}H-nmr spectrum of N,N'-(((oxybis(4,1-phenylene))bis(azanediyl))bis(carbonothioyl))dibenzamide **4**

……

(*Supplementary 9)*

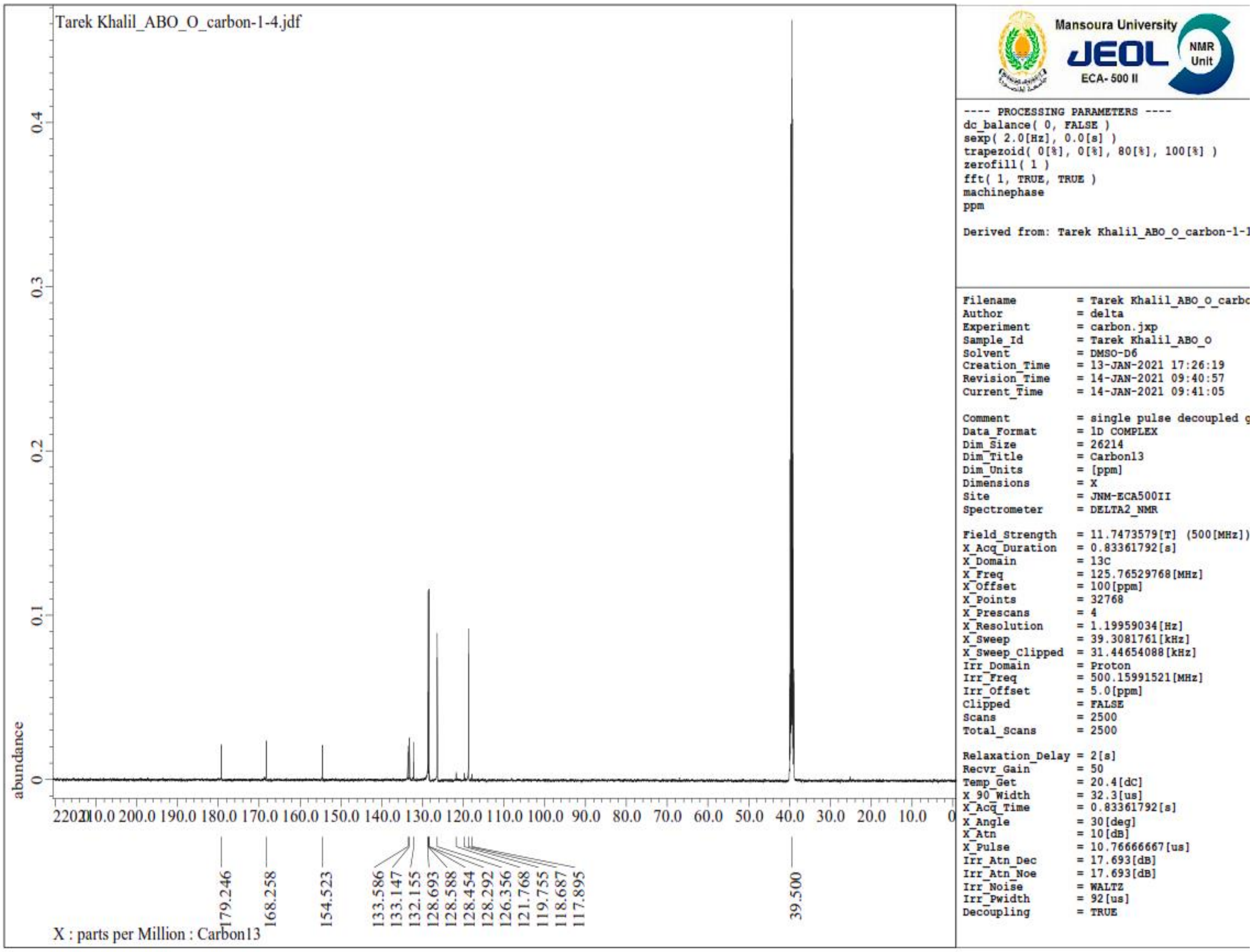

Figure S9: ^{13}C-nmr spectrum of N,N'-(((oxybis(4,1-phenylene))bis(azanediyl))bis(carbonothioyl))dibenzamide **4**

……

Supplementary 10

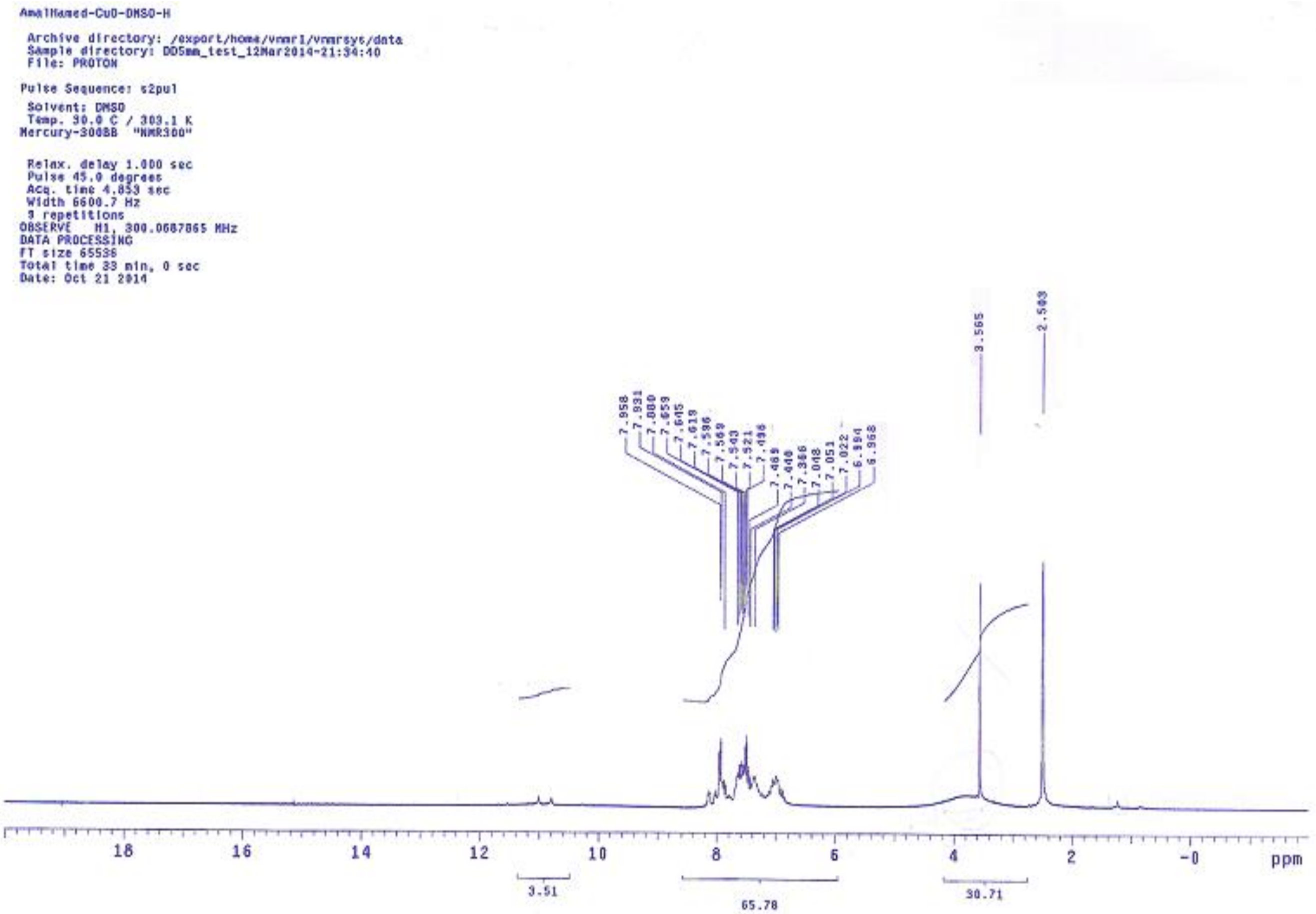

Figure S10: ^{1}H-nmr spectrum of copper complex **5**

……

Supplementary 11

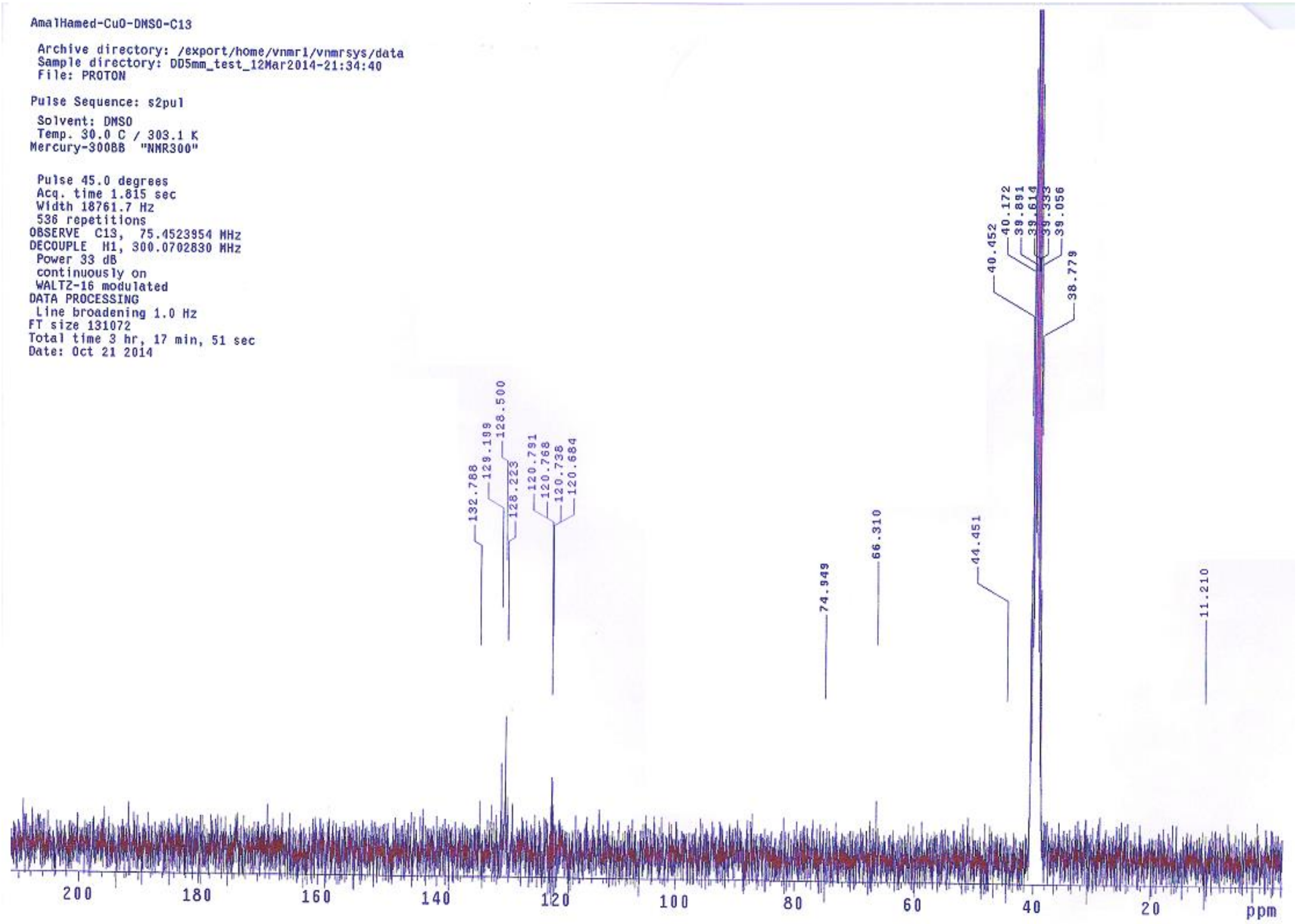

Figure S11: ^{13}C-nmr spectrum of copper complex **5**

………

Supplementary 12

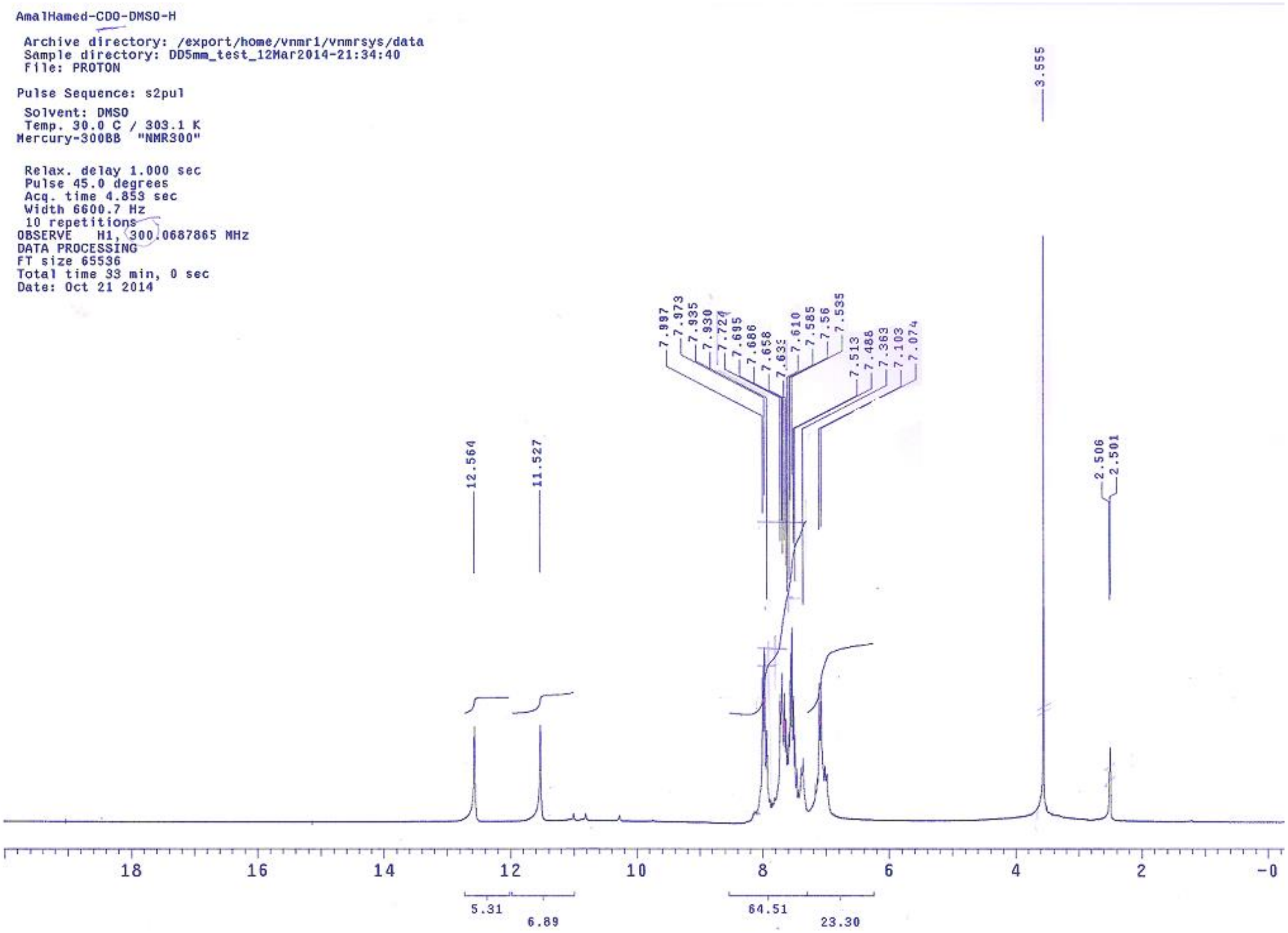

Figure S12: ^{1}H-nmr spectrum of cadmium complex **6**

……

Supplementary 13

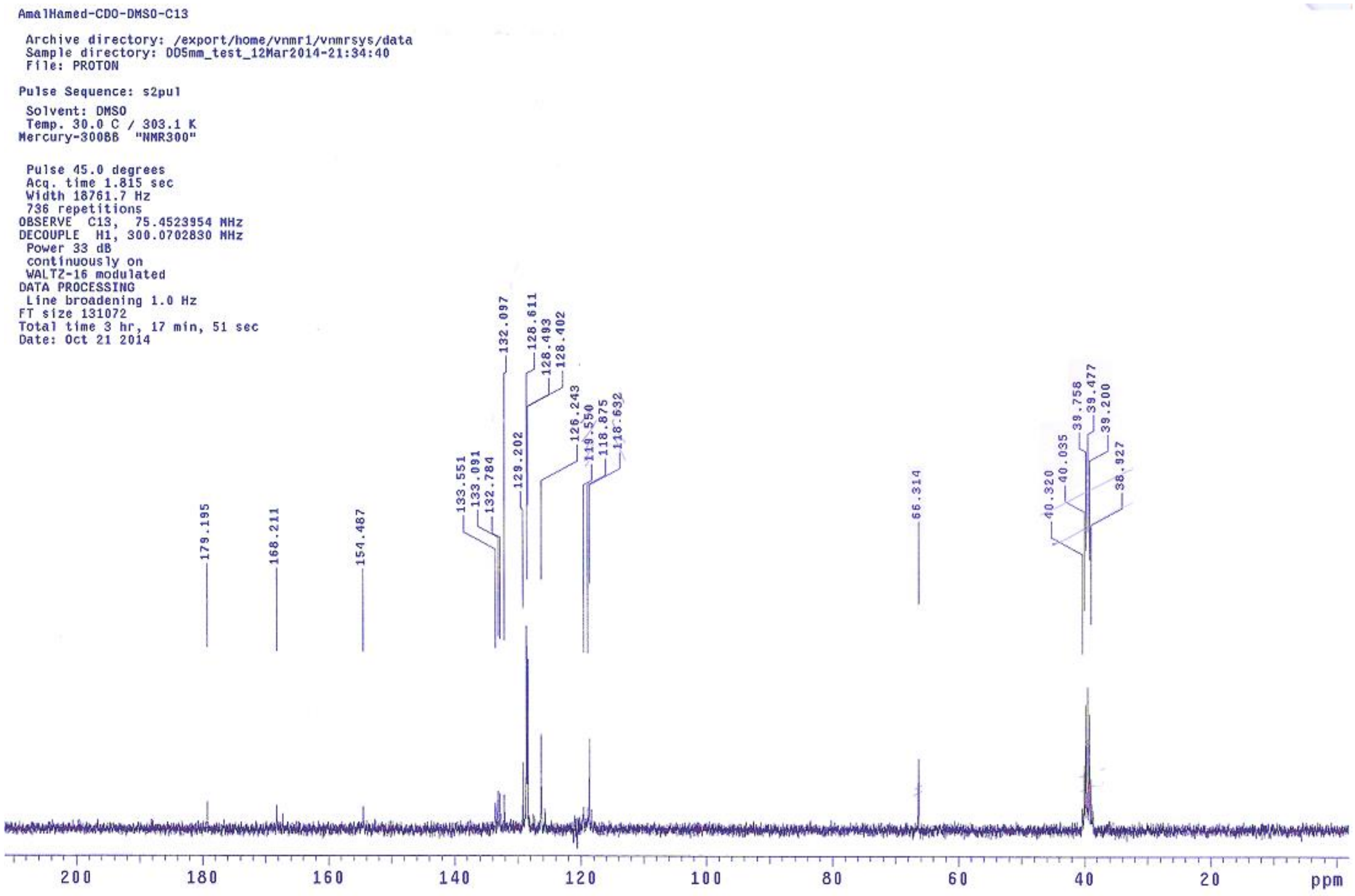

Figure S13: ^{13}C-nmr spectrum of cadmium complex **6**

....

Supplementary 14

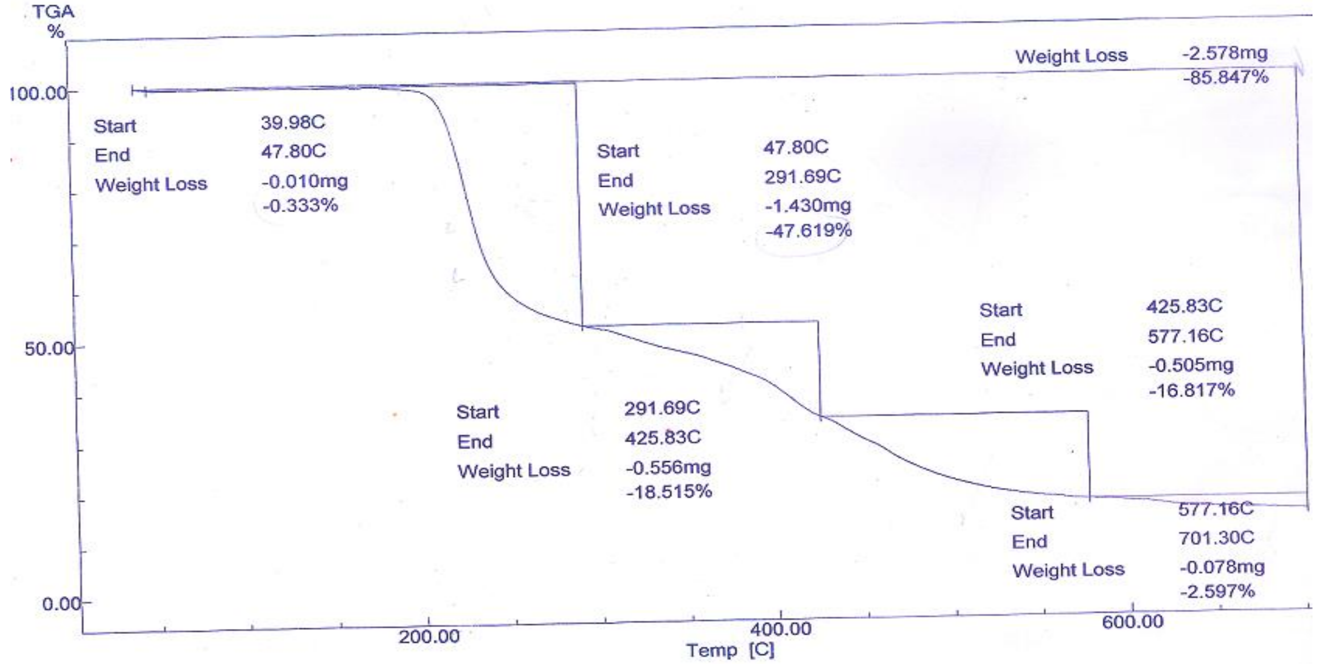

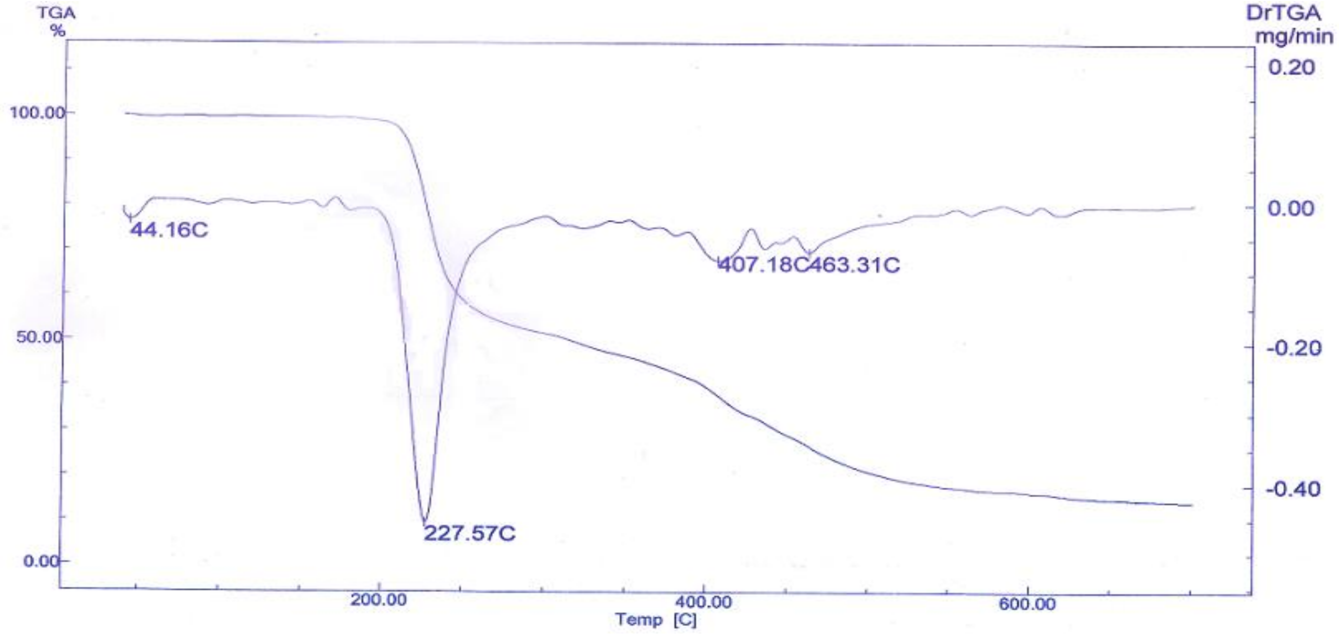

Figure S14: Thermal analysis data of N,N’-(((oxybis(4,1-phenylene))bis(azanediyl))bis(carbonothioyl))dibenzamide **4**

……….

Supplementary 15

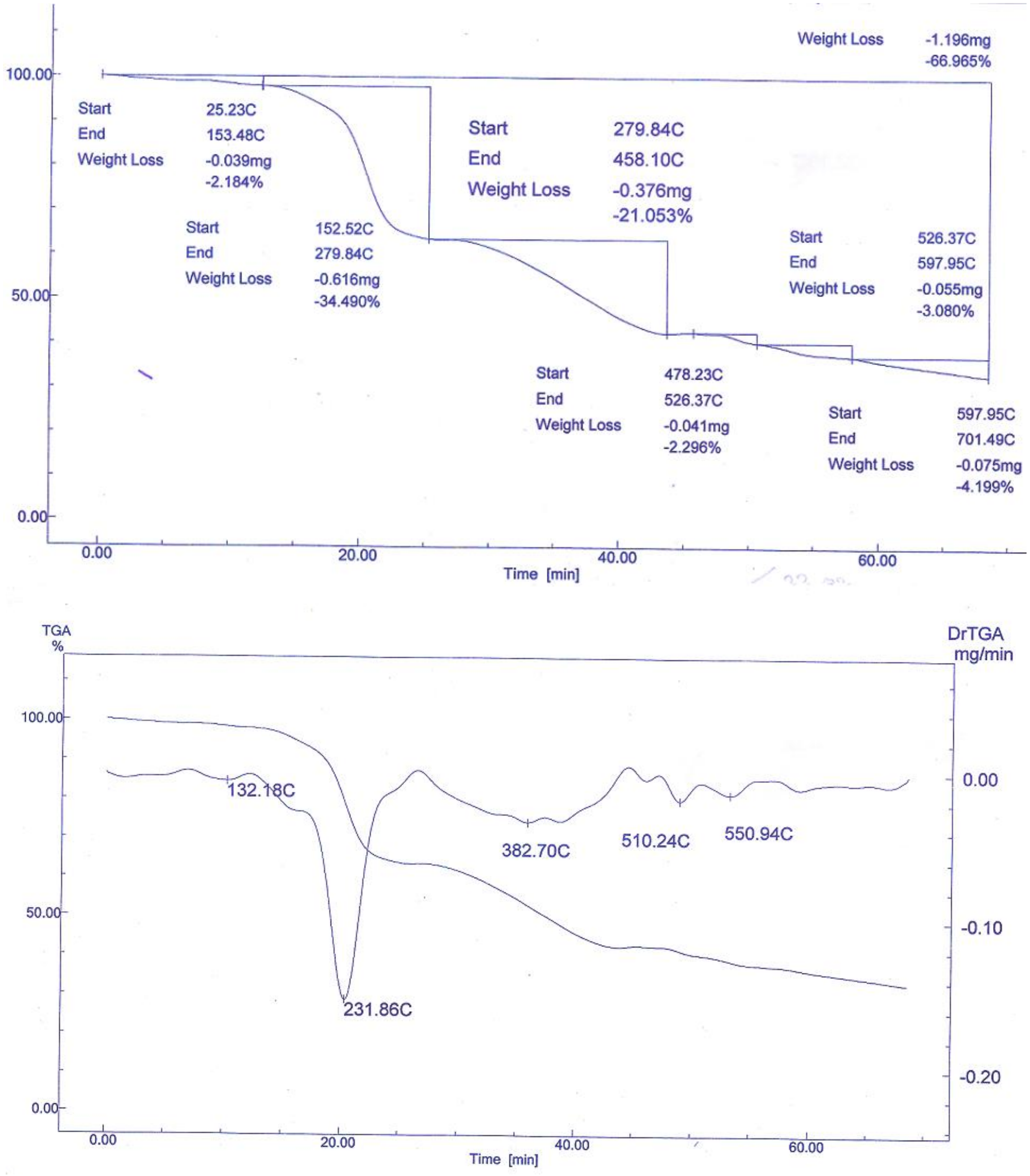

Figure S15: Thermal analysis data of copper complex **5**

………

Supplementary 16

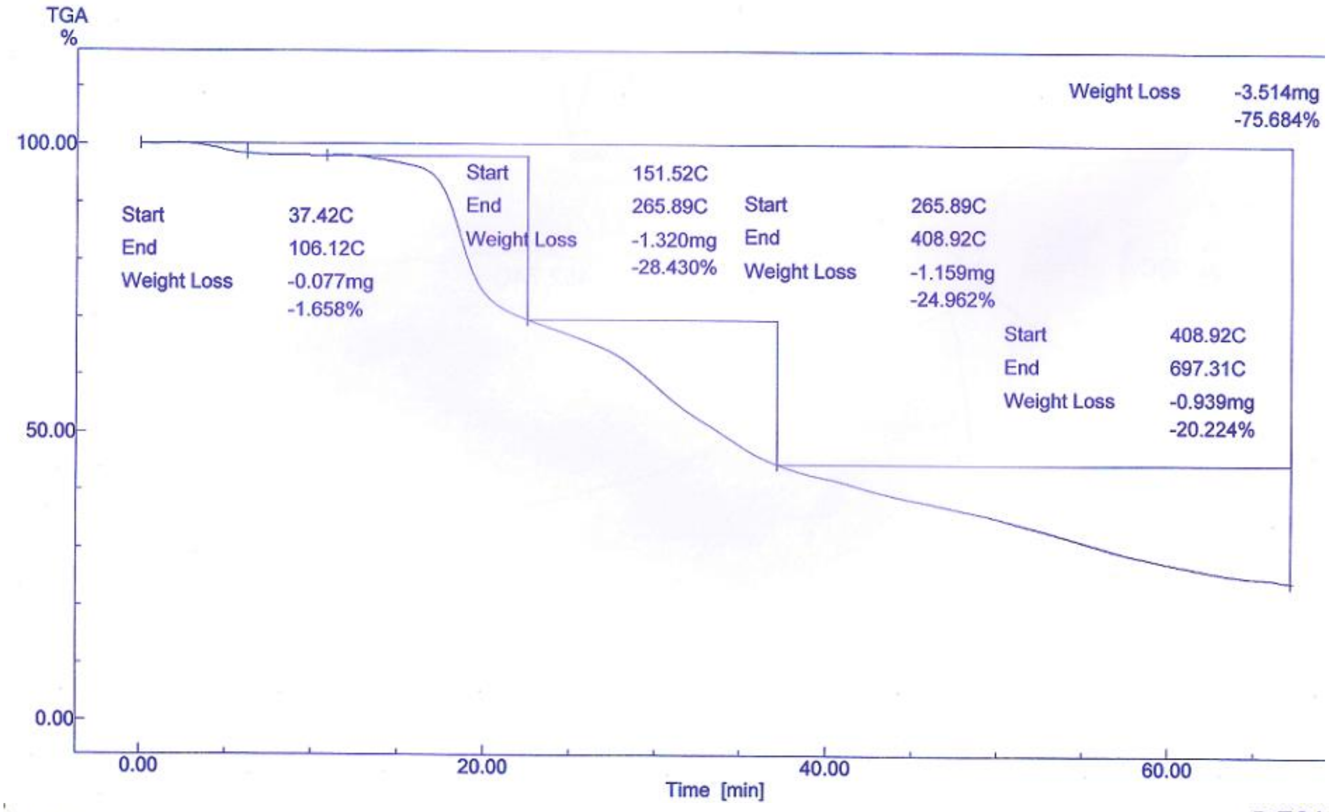

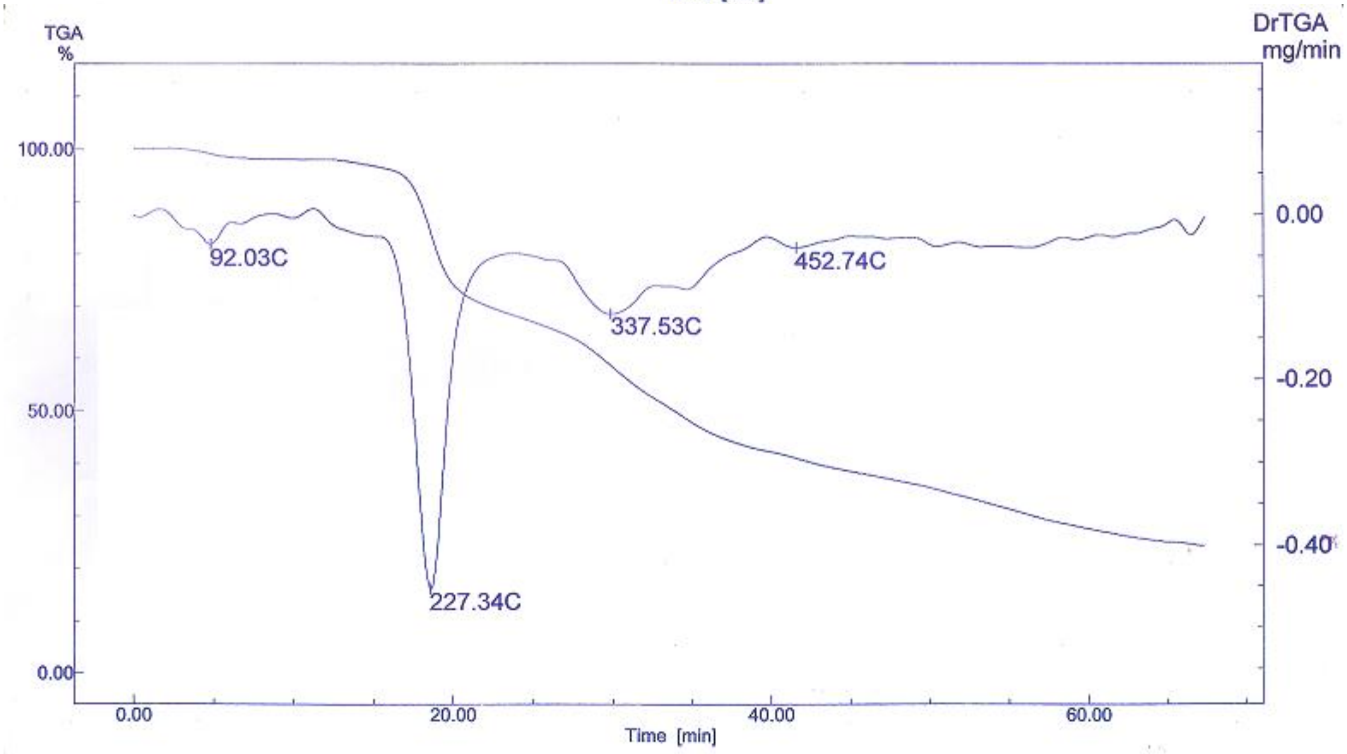

Figure S16: Thermal analysis data of cadmium complex **6**
